# *Mycobacterium abscessus* VapC5 toxin potentiates evasion of antibiotic killing by ribosome overproduction and activation of multiple resistance pathways

**DOI:** 10.1038/s41467-023-38844-4

**Published:** 2023-06-22

**Authors:** Eduardo A. Troian, Heather M. Maldonado, Unnati Chauhan, Valdir C. Barth, Nancy A. Woychik

**Affiliations:** 1 Department of Biochemistry and Molecular Biology, Rutgers University, Robert Wood Johnson Medical School, Piscataway, NJ 08854 USA; 2 Immunotherapy Laboratory, Basic Health Sciences Department, Federal University of Health Sciences of Porto Alegre (UFCSPA), R. Sarmento Leite, 245 - Centro Histórico, Porto Alegre, 90050-170 Brazil; 3 Member, Rutgers Cancer Institute of New Jersey, Piscataway, NJ USA

**Keywords:** Antimicrobial resistance, Pathogens, Proteomics, Transcriptomics, Ribosome

## Abstract

*Mycobacterium abscessus* (Mab) infections are inexplicably intractable to clearing after aggressive and lengthy treatment regimens. Here we discovered that acquisition of a single toxin-antitoxin system enables Mab to activate a phenotypic switch that enhances survival upon treatment with current first-line antibiotics. This switch is tripped when the VapC5 toxin inactivates tRNA^SerCGA^ by cleavage at only one site within its anticodon, leading to growth arrest. Concomitant tRNA^SerCGA^ depletion then reprograms the transcriptome to favor synthesis of proteins naturally low in the cognate Ser UCG codon including the transcription factor WhiB7 and members of its regulon as well as the ribosomal protein family. This programmed stockpiling of ribosomes is predicted to override the efficacy of ribosome-targeting antibiotics while the growth arrest phenotype attenuates antibiotics targeting cell wall synthesis. In agreement, VapC5 increases Mab persister formation upon exposure to amikacin and the next-generation oxazolidinone tedizolid (both target ribosomes) or cefoxitin (inhibits cell wall synthesis). These findings expand the repertoire of genetic adaptations harnessed by Mab to survive assaults intended to eradicate it, as well as provide a much-needed framework for selection of shorter and more efficacious alternate treatment options for Mab infections using currently available antimicrobials whose targets are not confounded by VapC5.

## Abstract (web summary)

*Mycobacterium abscessus* (Mab) infections are difficult to clear with antibiotics. Here the authors show that clinical Mab strains can acquire a toxin-antitoxin system that enhances survival upon treatment with current first-line antibiotics through depletion of tRNA^SerCGA^ and subsequent ribosome overproduction.

## Introduction

In the USA, the rapidly growing nontuberculous mycobacteria *Mycobacterium abscessus* is an emerging pathogen most recalcitrant to antibiotic clearing among the three strains comprising the *M. abscessus* complex: *M. abscessus* (referred to hereafter as “Mab”), *M. massiliense* and *M. bolletii*. Mab infections most commonly manifest as chronic lung disease or they attack skin and soft tissues^1–7^. Consequently, Mab infections take an especially high toll on individuals with cystic fibrosis. When inhaled, Mab can accelerate inflammatory lung damage in cystic fibrosis patients as well as cause serious pulmonary disease in the immunocompromised and other individuals with underlying lung disorders (reviewed in refs. ^1,8–11^). Modes of transmission are controversial, with some studies concluding that individuals with cystic fibrosis appear to contract Mab pulmonary infections via long-lived aerosols or fomites^12,13^ while a more recent study could not confirm cross-transmission among those with cystic fibrosis^14^. A 2021 phylogenomic analyses of a global set of Mab strains from patients with or without cystic fibrosis determined that global spread of Mab is not sequestered within the cystic fibrosis cohort^15^. A significant number of Mab infections are also acquired in the hospital, especially after plastic surgery, leading to skin and soft tissue infections. Mab skin infections can also occur upon exposure to contaminated syringes or needles. Nosocomial infections and suspected infections from fomites are difficult to prevent because Mab is resistant to most disinfectants and biocides^9^.

Mab infections display important similarities to certain features of *M. tuberculosis* infection, namely its ability to cause chronic disease associated with granuloma formation and to prolong infection by residing within phagocytes^3^. Yet, Mab is resistant to conventional antituberculars, and is generally one of the most drug-resistant mycobacteria. Compassionate use of bacteriophage therapy is the option of last resort for gravely ill patients with disseminated, antibiotic resistant Mab infections^16^. However, even bacteriophage therapy has limited efficacy because immunocompetent patients can generate neutralizing antibody within a few months after treatment^17^. There is an urgent need for better treatment options for Mab infections because even after treatment, patient prognosis is poor, and mortality remains high. Current treatment regimens typically last 1–2 years with an approximately 25–45% success rate^11,18–21^. It is not understood why Mab is so extraordinarily refractory to antimicrobial drugs. The difficulties in clearing Mab infections suggest that this bacterium enlists persistence to at least in part evade being killed when exposed to antibiotics. Persistence (often referred to as “intrinsic resistance” in Mab) refers to the ability of a bacterial pathogen to survive antibiotic exposure via a phenotypic switch, i.e., without acquisition of genetic mutations.

Activation of the toxin component of Type II toxin-antitoxin (TA) systems can facilitate antibiotic survival through persister cell formation, presumably because the action of the toxin places cells in a state of growth arrest to reduce the efficacy of antibiotics that act on actively growing cells^22–24^. Yet, the presence of TA systems in any of the three Mab complex subspecies has not been documented, nor has a role for TA systems been considered as a factor undermining the efficacy of treatment. TA systems are operons comprising adjacent genes encoding two small (~10 kDa) proteins, a toxin and its cognate antitoxin that inhibits toxin activity through formation of a stable TA protein-protein complex. Stress conditions are thought to lead to lower levels of the antitoxin and thus, a preponderance of free toxin which exerts its growth-regulating and/or other functions from within the bacterial cells^25^.

To investigate how TA systems might influence Mab physiology, virulence, and antibiotic susceptibility, we determined the mechanism of action of a representative VapC toxin, here designated VapC5 due to its similarity with the still uncharacterized VapC5 toxin from *M. tuberculosis*. Surprisingly, VapC5 not only tightly arrested growth, but also activated a circumscribed set of physiological changes that functionally align with antibiotic resistance. VapC5 dramatically upregulated the synthesis of the WhiB7 transcription factor and ribosomes to evade killing by amikacin and other ribosome targeting antibiotics. Overall, these studies led us to uncover a role for VapC5 in subverting the efficacy of first line Mab antibiotics without acquisition of one or more resistance mutations and lay the groundwork for evidence-based selection of alternate antibiotics whose potency is not abrogated by the action of this toxin.

## Results

### Many Mab clinical strains harbor TA systems

We searched the sequences of strains isolated from respiratory samples derived from Mab-infected patients that were archived in the NCBI genome database or the NCBI BioSample database (including whole-genome shotgun contigs). We identified 128 Mab strains that contained toxin genes orthologous to any of the toxin components for the ~90 annotated *M. tuberculosis* TA systems^26^. We found VapBC TA systems were most often represented in the clinical strains (the *M. tuberculosis* genome harbors 50 VapC TA toxins designated VapC1-VapC50^26^). Thirty two of the 128 clinical strains (25%) carried an apparent VapBC5 system, while the Mab reference strain ATCC 19977 does not carry this toxin.

**Fig. 1:**
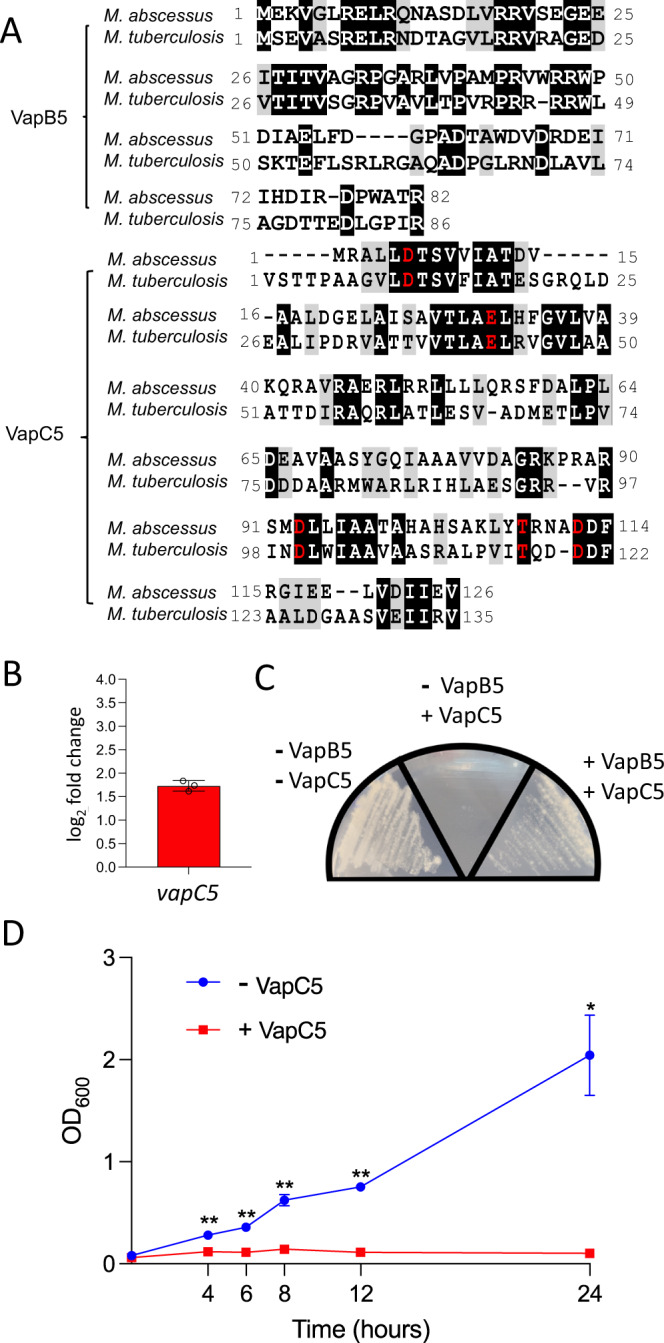
Many *M. abscessus* clinical strains contain an *M. tuberculosis* VapC5 ortholog whose expression leads to cell growth arrest. **A** T-Coffee and BoxShade alignment of Mab VapB5 and VapC5 to *M. tuberculosis* counterparts. **B** qPCR of Mab *vapC5*-pMC1s after 6 h of induction with ATc. **C** Mab cells containing both *vapC5*-pMC1s and *vapC5*-pNIT were streaked onto 7H9 plates containing either: no inducer, +ATc or +ATc +IVN (clockwise from left). **D** Mab cultures harboring either an empty vector (-VapC5, blue) or *vapC5*-pMC1s (+VapC5, red) were grown in 7H9 media until OD_600_ = ~0.1 followed by addition of ATc. Error bars represent standard deviation from the average of the three biological replicates. Asterisks represent statistical significance between control and induced in a two-sided Student’s *t*-test comparison (*p < 0.02; **p < 0.01). The raw data (**B** and **D**) or uncropped images (**C**) are in the Source Data file.

VapC (**V**irulence **a**ssociated **p**rotein) toxins are endoribonucleases that are only present in bacterial pathogens. In *M. tuberculosis*, VapC toxins predominantly cleave (and inactivate) specific tRNAs at a single site in the anticodon stem loop to generate tRNA-halves^27–34^. The Mab antitoxin VapB5 and toxin VapC5 were 47 and 52% similar, and 36 and 34% identical, to their counterparts in *M. tuberculosis*, respectively; Fig. 1A). This degree of conservation combined with the tandem, yet out of frame, orientation of the antitoxin and toxin genes strongly suggest that the Mab VapBC5 is a functional TA system. Therefore, in this work we investigated the detailed mechanism of action of the VapC5 toxin to determine if it contributed to Mab persistence upon antibiotic exposure.

### VapC5 expression leads to Mab cell growth arrest

The dynamic ratio of toxin:antitoxin is thought to impart reversible growth inhibition in response to stress, with the free toxin acting to downregulate growth^25^. To determine if the putative VapBC5 TA system behaves as predicted, the toxin alone or both toxin and antitoxin were coexpressed from plasmids in Mab ATCC 19977 cells (whose genome does not contain endogenous VapBC TA systems). Low level VapC5 expression (a modest 1.73 log_2_ fold change (3.31-fold) relative to the -VapC5 cells) under control of an anhydrotetracycline (ATc)-inducible promoter in the pMC1s plasmid (Fig. 1B) inhibited cell growth (middle quadrant, Fig. 1C), while coexpression of VapC5 with the VapB5 antitoxin from the isovaleronitrile (IVN)-inducible promoter of the pNIT plasmid rescued growth (right quadrant, Fig. 1C). VapC5-mediated growth arrest was evident as early as 4 h post-induction in liquid culture (Fig. 1D). Therefore, Mab VapBC5 behaves as a bona fide TA system.

### VapC5 preferentially targets tRNA^SerCGA^

VapC toxins are structure and sequence-specific endoribonucleases^35^. To identify the RNA target(s) of Mab VapC5, we used a specialized RNA-seq method, 5’ RNA-seq, developed in our laboratory^31,36^. 5’ RNA-seq identifies RNAs cleaved by VapC5 on a genome-scale and maps the toxin cleavage site to single nt resolution. VapC toxins generate a 5’ monophosphate (-P) upon cleavage of their RNA targets^27^. This 5’-P moiety cleanly distinguishes toxin-generated products from the majority of intact cellular RNAs.

**Fig. 2:**
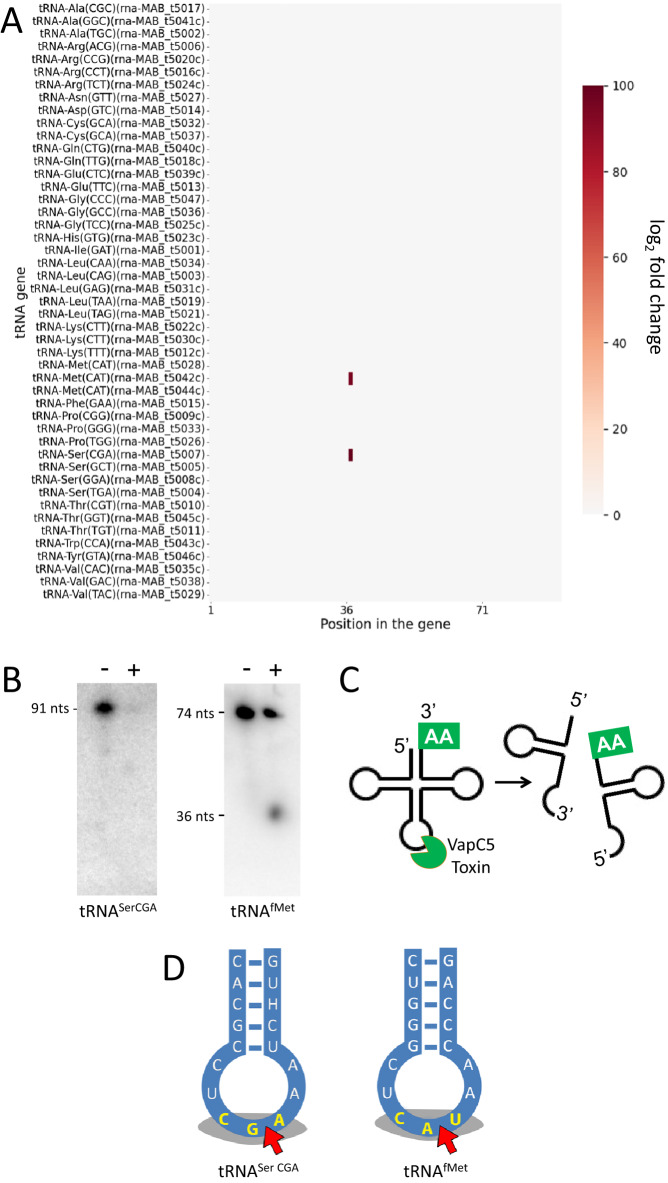
VapC5 targets two tRNAs within their anticodon in vivo. **A** Heatmap showing the fold changes of 5’ monophosphate levels (induced/uninduced) in each position of all 47 annotated Mab tRNA genes after 6 h of VapC5 induction; tRNA gene ID (from genome CU458896.1) shown in parentheses. Two additional tRNAs listed in the tRNA database^60^, but not annotated in the Mab reference genome, were also included in our analysis. Neither of these unannotated tRNAs with low (~35) general tRNA model scores—tRNA-Ile CAT (chr1.trna18) and tRNA Lys TTT (chr1.trna47)—were targeted by VapC5. Therefore, they are not listed in the heat map. **B** Northern analysis of VapC5 tRNA targets identified by 5’ RNA-seq. Each blot shown is representative of three independent experiments. Full length and positions of tRNA halves when visible are indicated. The oligonucleotide used for tRNA^SerCGA^ spans the cleavage site, precluding visualization of cleavage products. **C** Diagram of tRNA halves produced upon VapC5 cleavage. **D** Diagrams of Mab tRNA anticodon stem loops targeted by VapC5. Gray shaded nucleotides in yellow text, tRNA anticodons; red arrows, VapC5 cleavage sites identified by 5’ RNA-seq. The data source file for **A** is included as Supplementary Data 1 and the uncropped images for **B** are in the Source Data file.

We performed 5’-P RNA-seq on RNA harvested from Mab ATCC 19977 cells expressing VapC5 and identified two tRNAs specifically cleaved by this toxin: tRNA^SerCGA^ and to a lesser extent tRNA^fMet^ (Fig. 2A). These two tRNA targets were validated by northern analysis (Fig. 2B). As with other toxin tRNases, both were cleaved at a single site within their anticodon loop to generate tRNA halves (Fig. 2C)^27–34,37^. Figure 2D illustrates the sequence and structure of the two tRNA targets surrounding the VapC5 cut site. Notably, both tRNAs were cleaved between the 2nd and 3rd nt of the anticodon sequence. Overall, the identification of two tRNA targets was unexpected since the few examples of well characterized VapCs exhibit exquisite selectivity for a single tRNA in vivo^27–30,34,38^.

### VapC5 tRNA targets share sequence and structural features

The selectivity of VapC toxins for their tRNA targets appears to require both sequence and structural determinants^30,31^. Therefore, we assessed the sequence similarities between the two full length tRNAs cleaved by Mab VapC5 and identified 20 identical nts dispersed throughout their sequences (Supplementary Fig. 1A). However, sequence conservation was concentrated within the regions near the 5’ end through the variable stem loop moving 5’ to 3’ along the tRNA (Supplementary Fig. 1B). Collectively, these conserved regions comprise the vertical arm of the upside-down L-shaped tRNA tertiary structure. This arm of tRNA is predicted to interact with VapC dimers based on our published docking simulations of an *M. tuberculosis* VapC with tRNA^31^. In addition, recognition of determinants in the sugar phosphate backbone likely contribute to cleavage specificity of VapC5 and other VapC toxins because aminoacylases recognize determinants in the tRNA sugar phosphate to ensure correct amino acid attachment to tRNA^39^. Finally, tRNAs can also be post-transcriptionally modified and the presence, or lack of, a particular modification may influence recognition by VapC5. In summary, tRNA sequence, structure, modifications, and sugar phosphate backbone are all predicted to enable VapC5 to distinguish two tRNAs from among the 47 Mab tRNAs and other highly structured RNA species in vivo.

### VapC5 strongly upregulates *whiB7*

**Fig. 3:**
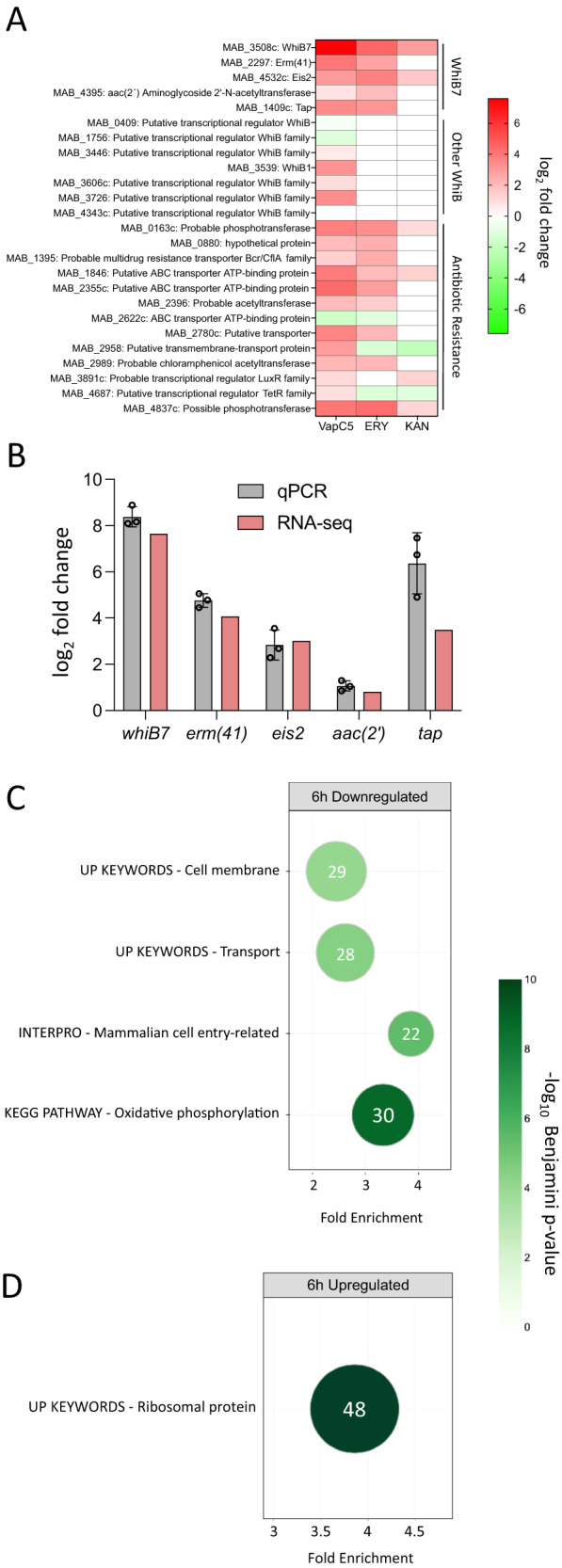
VapC5 induces the expression of WhiB7, ribosomal proteins and multiple antibiotic response genes. **A** Heat map of transcript changes in 25 genes associated with Mab antibiotic resistance in ATCC 19977 upon VapC5 expression (“VapC”, left column; all adjusted *p* < 0.0001), erythromycin treatment (“ERY”, middle column) or kanamycin treatment (“KAN” right column) from Miranda-CasoLeuengo et al.^44^ “WhiB7” group, Mab transcripts in WhiB7 regulon associated with antibiotic resistance. “Other WhiB” group, other Mab WhiB family annotated transcripts. “Antibiotic Response”, the 13 Mab designated antibiotic response transcripts from Miranda-CasoLuengo et al.^44^. White boxes in ERY and KAN columns denote no differential expression for the 13 antibiotic responsive genes or not present in the RNA-seq data set for the seven “Other WhiB” transcripts. The data source file is included as Supplementary Data 2. **B** qPCR validation of top five RNA-seq WhiB7-related transcripts shown in **A**. Data derived from three biological samples with three technical replicates for each biological sample. Error bar represents standard deviation. **C**, **D**. DAVID functional analysis^45,46^ gene categories significantly (≥ 2-fold) down- (**C**) or upregulated (**D**) 6 h after VapC5 induction. The area of each circle in both panels is proportional to the number of observed transcripts in the corresponding category; transcript number also shown within each circle. Cohen’s kappa statistical test with Benjamini correction used for **C**, **D**. The data source file for **C**, **D** is included as Supplementary Data 3. The raw data for **B** is located in the Source Data file.

Genome-wide comparison of the abundance of transcripts from cells with or without VapC5 expression by RNA-seq revealed that the *whiB7* transcript exhibited the highest increase in expression in the entire 4971-transcript dataset in two independent experiments each with multiple replicates (*first transcript*, Fig. 3A; Supplementary Data 2). The *whiB7* transcript is known to be upregulated by several ribosome-targeting antibiotics in Mab^40–42^. WhiB7 also activates the ribosomal methylase Erm(41) that contributes to intrinsic macrolide resistance in Mab^43^. The *erm*(41) gene was significantly upregulated in our RNA-seq datasets (*2*nd *transcript*, Fig. 3A). Transcripts encoding the Gcn5-related *N*-acetyltransferase *eis2*, the aminoglycoside 2´-*N*-acetyltransferase *aac(2’)* and the efflux pump *tap* are also upregulated by WhiB7 (*3rd, 4th, and 5th* *transcript*, Fig. 3A)^40,41,44^. The upregulation of *whiB7, erm*(41), *eis2, aac(2’)* and *tap* upon VapC5 expression was validated by qPCR (Fig. 3B).

Mab has seven other WhiB family transcription factors whose functions are not defined, only some of which are also significantly upregulated by VapC5 (“Other WhiB”, Fig. 3A). The effect of VapC5 on the transcription of 13 antibiotic response genes identified by Miranda-CasoLuengo et al. through RNA-seq of erythromycin or kanamycin treated Mab ATCC 19977 is shown in the “Antibiotic Response” category in Fig. 3A^44^. The expression profile of these 13 genes is completely distinct, and less robust, than that of VapC5 (Fig. 3A).

We next used the Database for Annotation, Visualization and Integrated Discovery (DAVID)^45,46^ to help define other statistically significant up- and down-regulated functional categories of transcripts from the RNA-seq dataset that were not as readily recognizable as WhiB7 (Supplementary Data 3). The theme within the downregulated gene categories was consistent with cells in a state of growth arrest, e.g., reduced transport (many shared with the ‘cell membrane’ category) and energy production (Fig. 3C). Genes for mammalian cell entry were also downregulated, suggesting a role for VapC5 in other stages of infection or Mab lifecycle. By contrast, there was only one significantly upregulated category: ‘ribosomal proteins’, comprising 48 of the 58 ribosomal protein genes (Fig. 3D). In fact, nearly all were upregulated to some extent but 48 met our stringent cut offs for expression level and statistical significance. Clearly, VapC5 selectively orchestrates upregulation of ribosomal protein genes.

### VapC5 expressing cells stockpile ribosomes

**Fig. 4:**
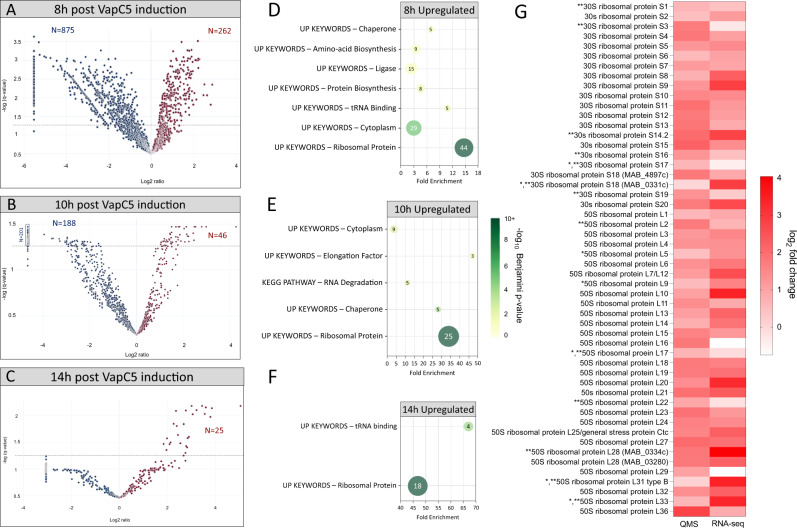
VapC5 predominantly upregulates the synthesis of ribosomal proteins. **A**–**C** ±VapC5 cultures were grown to an OD of 0.1, ATc added for 6 h, 8 h or 12 h followed by AHA-labeling for 2 h (*n* = 4) resulting in overall ATc induction times of 8, 10 and 14 h. Newly synthesized AHA-labeled proteins represented as volcano plots. Blue, down regulated proteins; red, upregulated proteins. Horizontal dotted line indicates significance cut-off (-log *q* = 1.3). **D**–**F** DAVID Functional Analysis Tool terms^45,46^ associated with proteins significantly upregulated (Strimmer *q* ≤ 0.05) following 8 h, 10 h or 14 h of VapC5 induction. Circle color corresponds to its -log10 Benjamini *p*-value, circle diameter reflects the relative number of proteins in the corresponding category; protein number also indicated within each circle. **G** Heatmap showing concordance between the upregulation of ribosomal protein transcripts (RNA-seq; 6 h VapC induction) and upregulation of new ribosomal protein synthesis (quantitative mass spectrometry, QMS; 8 h VapC induction). One asterisk, upregulated in QMS but Strimmer *q* > 0.05. Two asterisks, upregulated in RNA-seq but *p* > 0.05. 50 S ribosomal protein L28 and 30 S ribosomal protein S18 are translated from two different mRNAs. Of 58 ribosomal genes (and 57 ribosomal proteins), only 52 were present in both datasets and were shown. The data source file for **A**–**C** is included as Supplementary Data 4; **D**–**F**, Supplementary Data 5, **G** Supplementary Data files 2 and 4. The statistical test used for **D**–**F**, Cohen’s kappa statistical test with Benjamini correction.

We next tracked how VapC5 influences the Mab proteome. Mab was metabolically labeled with the azide-containing Met mimetic azidohomoalanine (AHA) and newly synthesized proteins were captured on alkyne-coated agarose beads using click chemistry. Quantitative mass spectrometry enabled genome scale identification of proteins whose synthesis was up- or down-regulated after 8, 10, or 14 h of VapC5 expression relative to the empty vector control (Fig. 4, Supplementary Data 4). We detected over a thousand proteins whose synthesis was downregulated after VapC5 expression (Fig. 4A–C). However, as protein synthesis in these growth arrested cells diminished after extended toxin induction, fewer downregulated proteins met our statistical cutoff. Among the 1063 statistically significant downregulated proteins, 875 proteins were downregulated after 8 h (Fig. 4A) and 188 were downregulated after 10 h (Fig. 4B); no downregulated proteins met the statistically significant threshold after 14 h (Fig. 4C). DAVID analysis of the downregulated proteins in the 8 and 10 h datasets placed them in functional categories consistent with cells in a state of growth arrest, e.g., peptidoglycan synthesis, DNA replication, energy production and metabolism (Supplementary Data 5, down-regulated tab).

We also identified hundreds of proteins whose synthesis increased relative to the control upon VapC5 expression (Fig. 4D–F) even though cells were very tightly growth arrested (Fig. 1D). DAVID analysis revealed that these proteins fall into a surprisingly small number of interrelated functional categories with ‘ribosomal protein’ as the dominant theme (Fig. 4D–F, Supplementary Data 5, up-regulated tab). Many of the same proteins fall into one or more of the categories. First, there was sustained new synthesis of ribosomal proteins 8, 10, and 14 h after VapC5 expression (Fig. 4D–F). Of the 57 ribosomal proteins encoded by 58 genes (two genes encode ribosomal protein S18) in Mab, all but four very small ribosomal proteins were collectively detected in the three datasets. The four missing ribosomal proteins—L35, L34, L31, and L30 that are only 64, 47, 74, and 60 amino acids long, respectively—generate too few tryptic peptides of the length needed for reliable detection by mass spectrometry. The ‘tRNA-binding’ group contains five ribosomal proteins that interact with tRNA; these five are also represented in the ‘ribosomal protein’ category (Fig. 4F).

All other essential components of the basic protein synthesis machinery, i.e., initiation factors IF-1, IF-2, and IF-3 as well as elongation factors EF-Tu, EF-Ts, EF-G, and EF-P, were detected by mass spectrometry and upregulated in the newly synthesized protein pool (represented within the ‘protein biosynthesis’ and/or ‘cytoplasm’ DAVID categories, Fig. 4D). Although IF-2 and EF-P were detected at levels above the control (~0.4 log_2_ fold change), they were below our ±0.58 log_2_ fold change cut off (i.e., ±1.5 fold) and excluded from the dataset used for DAVID analysis. Since GTP-bound IF-2 binds to tRNA^fMet^ and brings it to the initiation complex, VapC5-induced depletion of tRNA^fMet^ may result in coordinate signals that reduce the levels of available IF-2. EF-P is engaged only in special cases, runs of prolines residues, so may not need to be as abundant at the others. The initiation and elongation factors only transiently interact with ribosomes in the initiation or elongation complex and are continuously recycled. Therefore, they are not expected to be needed at levels as high as ribosomal proteins.

In the ‘chaperone’ category, GroEL, GroES, trigger factor, DnaJ1 and DnaJ2 are significantly upregulated 10 h after VapC5 expression (Fig. 4E). Although DnaK (Hsp70) is also significantly upregulated at all time points (0.81, 1.84, and 1.64 log_2_ fold change, respectively), the 8 h and 10 h strimmer *q*-values did not meet our statistical cut off (≤0.05) for inclusion in DAVID analysis. Coordinate synthesis of chaperones with ribosomal proteins is consistent with their roles in ribosome assembly and protein synthesis^47^. Trigger factor directly associates with ribosomes to promote co-translational folding of nascent polypeptides while other chaperones aid in this folding process^48^. Many of these chaperones fall into the ‘RNA degradation’ category as well because they participate in rRNA maturation^47^ (Fig. 4E). As the length of VapC5 induction increased to 14 h, just two DAVID categories remained: ‘ribosomal protein’ and ‘tRNA-binding’ (Fig. 4F). However, both categories contain only ribosomal proteins; four ribosomal proteins interact with tRNA and also sort to the ‘tRNA-binding’ category. Therefore, widespread upregulation of genes encoding ribosomal proteins (Fig. 3D) was followed by comprehensive upregulation of ribosomal protein synthesis as well (Fig. 4G).

Although not represented in the DAVID analysis because many of the corresponding genes are not annotated, we discovered that nearly all enzymes in the TCA cycle are upregulated 8 h post induction. This may result in an initial burst of energy that generates ATP and NADH as well as amino acid precursors that are functionally integrated with the 8 h ‘amino acid biosynthesis’ DAVID category. Several tRNA synthetases sort to the 8 h ‘ligase’ subset along with a few unrelated metabolic enzymes with ligase activity (Fig. 4D). In summary, the predominant functional themes among newly synthesized proteins upregulated upon VapC5 expression center on ribosome biosynthesis, translation factors and energy production.

Finally, we examined the ±VapC5 quantitative mass spectrometry datasets for the transcriptional activator WhiB7 and the proteins encoded by its four well characterized target genes (*first five genes in* Fig. 3A): Erm(41) ribosomal methylase, Eis2 N-acetyltransferase, Aac(2’) aminoglycoside 2’-*N*-acetyltransferase and Tap efflux pump. Note that proteins can only be detected by AHA proteomics if they contain an internal Met—WhiB7 and Erm(41) do not—and if we performed mass spectrometry on the fraction it resides in. Therefore, because we did not enrich for the membrane protein fraction, our ability to detect Tap is hampered. The two proteins that should be detectable, the cytoplasmic proteins Aac(2’) and Eis2, were detected, upregulated, and statistically significant. New synthesis of Aac(2’) increased 3.3-fold and new synthesis of Eis2 protein was up 1.7 fold following VapC5 expression (Supplementary Data 4, 8 h tab).

### tRNA^SerCGA^ depletion leads to codon-dependent proteome remodeling

**Fig. 5:**
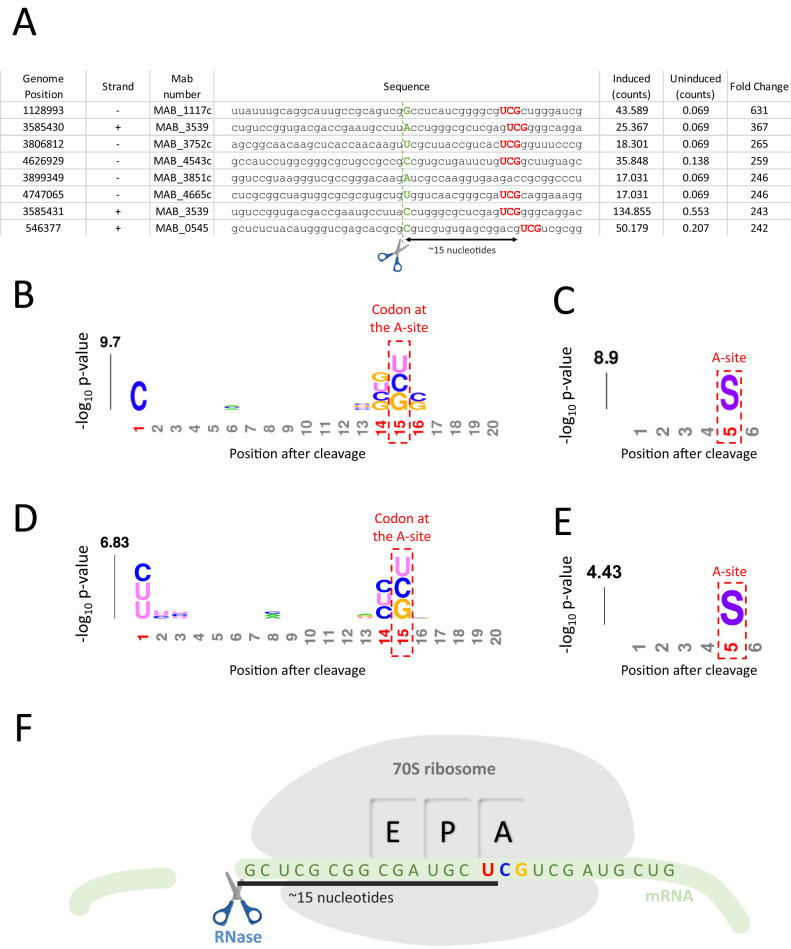
Depletion of tRNA^SerCGA^ by VapC5 leads to ribosome stalling at Ser UCG codons. **A** Top 8 Mab mRNA hits in our 5’ RNA-seq dataset illustrate Ser UCG codons (red) ∼15 nucleotides downstream of the RNase cleavage site 5’ of the stalled ribosome (green dotted line; compare to illustration in **F**). The genome position and strand where the secondary cleavage occurs is shown as well as the MAB number of the gene containing the Ser codon. **B** *k*pLogo^62^ constructed from the top 100 mRNAs identified by 5’ RNA-seq in +VapC5 Mab cells. Significantly enriched 3-mers (adjusted *p* ≤ 0.05) are shown; numbered positions colored in red. Positions are numbered relative to the cleavage site; predicted ribosome A-site is indicated, ~15 nts after green dotted cleavage site shown in **A**. **C** Amino acid *k*pLogo^62^ constructed from the top 100 hits of the coding sequences identified by 5’ RNA-seq in +VapC5 Mab cells. Only significant (adjusted *p* ≤ 0.05) enriched amino acids are shown at each position. Amino acids are represented in their single letter code. Positions are numbered relative to the predicted cleavage site and the ribosome A-site is indicated at position 5 (~15 nts after green dotted cleavage site shown in **A**). **D**, **E** Nucleotide and amino acid *k*pLogos derived from 5’ RNA-seq of VapC5 expressed in *M. smegmatis*. Significantly enriched *k*pLogo 3-mers (adjusted *p* ≤ 0.05) are shown; numbered position colored in red in **D**. Amino acid *k*pLogo constructed from the top 100 mRNA hits. Only significant (adjusted *p* ≤ 0.05) enriched amino acids are shown at each position in **E**. **F** Illustration of mRNA stalled at hungry Ser UCG codon at the A-site showing cleavage of the transcript by the unspecified recycling RNase ~15 nts upstream. The data source files for **B**, **D** are included as Supplementary Data files 1 and 6, respectively. The statistical test used for **B**–**E**, unweighted binomial test with Bonferroni correction.

To better understand how VapC5 executes such circumscribed effects on Mab physiology upon selective inactivation tRNA^SerCGA^ we first looked for ribosome stalling at Ser UCG codons. We have previously demonstrated in both *M. tuberculosis* and *M. smegmatis* that genome-wide ribosome stalling can be detected and precisely mapped within 5’ RNA-seq datasets for tRNase toxins that cleave and inactivate a single elongator tRNA^27,28,38^. In perfect agreement, we documented the same effect for VapC5, i.e., depletion of tRNA^SerCGA^ leads to ribosome stalling at Ser UCG codons in both Mab (Fig. 5A–C) and M. *smegmatis* (Fig. 5D, E). Each stalling event is readily detected because there is a conspicuous ~15 nt distance in the relevant RNA-seq dataset from the RNA cleavage site to an in-frame Ser UCG codon requiring the depleted tRNA (Fig. 5A). In Barth et al., we used Ribo-seq to prove that this ~15 nt distance corresponds to the footprint of the stalled ribosome on mRNA due to the “hungry” codon at the A-site (Fig. 5F)^38^. We fortuitously detect these stalled ribosomes because the mRNAs that harbor them are recycled by an RNase distinct from VapC5 that cuts the transcript and leaves a characteristic 5’-moiety detectable by 5’ RNA-seq (Fig. 5F).

**Fig. 6:**
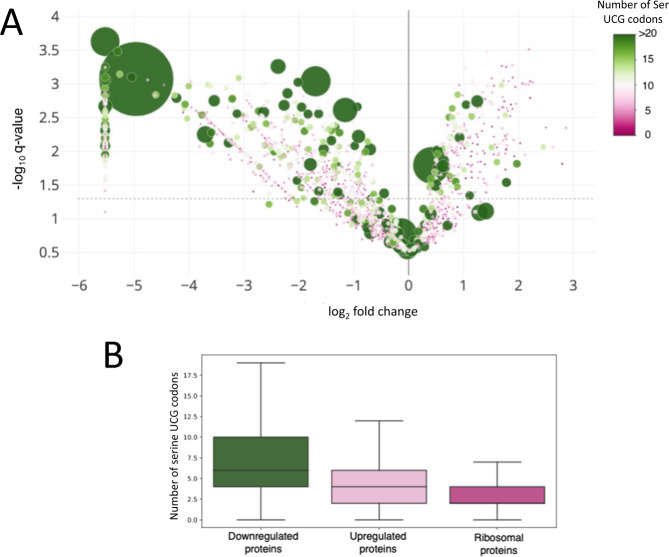
VapC5 expression leads to codon-dependent shifts in translation. **A** Volcano plot showing the changes (log_2_ of fold changes) in newly synthesized proteins between +VapC5 versus -VapC5 Mab cells. The gray dotted line marks the statistical cutoff (Strimmer *q* ≤ 0.05). The diameter and color of each represented protein (circle) reflects the number of UCG codons in its coding sequence. The source data are presented in Supplementary Data 4. **B** Box plot depicting the distribution of Ser UCG codons in the coding sequences of the significantly up- or downregulated proteins (statistical cutoff at log_2_ fold change ≥ +/−1 and Strimmer *q* ≤ 0.05) as well as upregulated ribosomal proteins in the AHA-labeled proteomics dataset. Outliers are not shown. The number of proteins in each category: 875 downregulated proteins, 262 upregulated proteins, 45 ribosomal proteins.

**Fig. 7:**
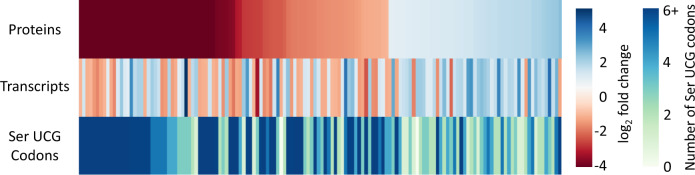
mRNA levels correlate with protein expression in VapC5 expressing Mab cells. Heatmaps comparing newly synthesized proteins detected by quantitative mass spectrometry (Proteins) to mRNA levels via RNA-seq (Transcripts) to the number of UCG codons in each transcript in ±VapC5 cells. Transcripts/proteins with a log_2_ fold change of ±1 were considered. Source data are provided as a tab within Supplementary Data files 2 and 4.

The single cleavage site before one or more stalled ribosomes at Ser UCG codons is expected to result in a truncated, nonfunctional mRNA. This, in turn, should result in a decrease in the abundance of the proteins encoded by these transcripts. We plotted the 8 h quantitative mass spectrometry data used in Fig. 4 as a volcano plot with individual proteins represented as a colored circle whose diameter and color are scaled to reflect Ser UCG codon content (Fig. 6A). The effect of Ser UCG content on the abundance of newly synthesized proteins is graphed as a box whisker plot showing that downregulated proteins tend to have a higher Ser UCG codon content than upregulated proteins (Fig. 6B). We also plotted upregulated ribosomal proteins for Ser UCG content. In agreement, ribosomal proteins contained fewer Ser UCG codons (Fig. 6B). Comparison of Ser UCG content, fold-changes in proteins, and fold-changes in transcripts demonstrated that the anticipated trends were maintained genome-wide. VapC5 mediated the global reduction in Ser UCG-codon containing transcripts and newly synthesized proteins (Fig. 7).

### VapC5 enhances persister formation

**Fig. 8:**
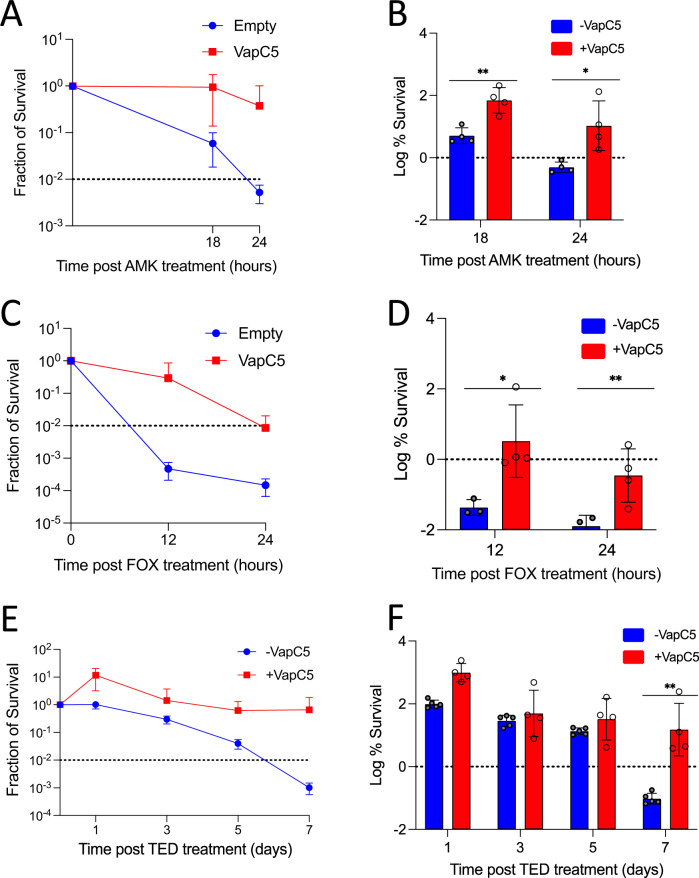
VapC5 potentiates intrinsic resistance in Mab. +VapC5 (red) or -VapC5 (empty vector, blue) expressing cells were exposed to either 10X MIC for amikacin (AMK) and cefoxitin (FOX), or 5X MIC for tedizolid (TED). Survival was compared to the empty vector control (-VapC5). Error bars indicate standard deviation. Asterisks represent statistical significance between control and induced in a Student’s t-test comparison, paired, one-tailed (*p ≤ 0.05, **p ≤ 0.007). The data source file is included as Supplementary Data 7. **A**, **B** four replicates for each group; **C**, **D** four replicates for VapC5 and three replicates for empty; **E**, **F** four replicates for VapC5 and five replicates for empty.

The upregulated proteins identified by quantitative mass spectrometry revealed valuable clues to how VapC5 manipulates Mab physiology to favor survival during antibiotic treatment. Therefore, we first tested whether VapC5 influences cell survival after treatment with the aminoglycoside amikacin or the cephalosporin cefoxitin, first line antibiotics for treatment of Mab pulmonary disease^49^. Mab cells containing VapC5 or empty vector were exposed to 10X the minimal inhibitory concentration (MIC) of amikacin or cefoxitin for up to 24 h, serially diluted and plated to determine CFU/ml. It is recommended to use at least several times MIC to ensure that one is measuring true persistence and not transient modes of resistance such as heteroresistance^50^; it is also important to perform a kill curve with ±VapC5 cells to ensure that -VapC5 control cells reach the minimum duration to kill 99% of the population (MDK_99_). Comparison of the killing curves for each antibiotic tested revealed that each -VapC5 control reached the MDK_99_ while +VapC5 cells did not (Fig. 8A, C, E). We observed marked increases in recovery of viable cells after 24 h of amikacin or cefoxitin exposure in VapC5 expressing cells compared to the control (Fig. 8A–D). We next tested tedizolid, an oxazolidinone with a lower MIC, high bioavailability and a higher safety profile than the other antibiotic in this class, linezolid, used as an alternative treatment for Mab infections^21,49^. Both linezolid and tedizolid exhibit low frequencies of spontaneous resistance^21^. VapC5 expression also promoted persister formation with tedozolid (Fig. 8E, F). These results indicate that the killing action of amikacin, cefoxitin, and tedizolid is substantially weakened when VapC5 is expressed, leading to a larger pool of persisters that can seed growth once the antibiotic is removed. This effect occurs without acquisition of other antibiotic resistance genes or mutations in antibiotic targets.

Mechanistically, amikacin inhibits protein synthesis by irreversibly binding to 16 S rRNA and the S12 ribosomal protein components of the 30 S subunit while tedizolid inhibits bacterial protein synthesis by binding to 23 S rRNA of the 50 S subunit. In alignment with the activity of amikacin, our quantitative mass spectrometry data shows a VapC5-mediated burst in synthesis of the S12 ribosomal protein 8 h after toxin induction (1.76 log_2_ fold change = 3.4-fold change). This newly synthesized S12 protein is produced along with the other ribosomal proteins as shown in Fig. 4G. This large pool of newly synthesized ribosomal proteins is expected to assemble into stable Mab ribosomes and build the cache of ribosomes predicted to collaborate in confounding the efficacy not only amikacin, but tedizolid as well.

## Discussion

**Fig. 9:**
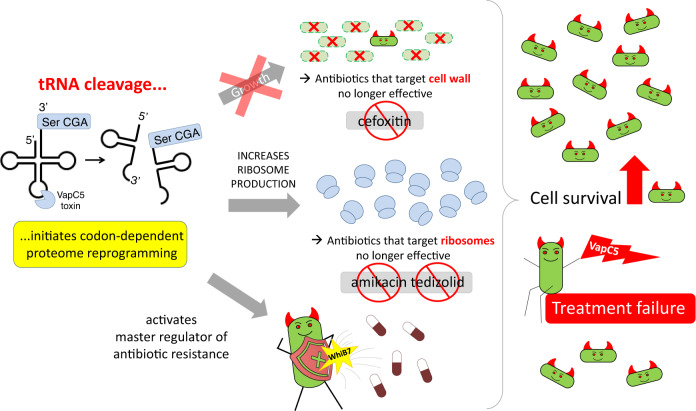
Mab VapC5 toxin subverts the efficacy of first line Mab antibiotics by activating multiple resistance pathways. The VapC5 endonuclease TA toxin primarily cleaves elongator tRNA^SerCGA^. Inactivation and depletion of tRNA^SerCGA^ triggers transcriptional changes that in turn remodel the proteome toward the robust, sustained and nearly exclusive synthesis of ribosomes and other components of the translation machinery even though cells are in a state of complete growth arrest. First, the strong cell growth arrest phenotype characteristic of VapC5 should dampen the efficacy of all antibiotics that require actively growing cells (e.g., amikacin, cefoxitin, and tedizolid). Second, the overproduction of ribosomes and translation factors builds a strategic reserve of translational capacity that overrides the efficacy of all antibiotics that target ribosomes and helps cells recover when antibiotics are removed. Third, dramatic upregulation of WhiB7 and its targets collaborate to resist the action of multiple antibiotics, resulting in Mab survival, and clinically, as treatment failure.

Mab is ubiquitous in the environment. Therefore, clinical isolates harboring VapC5 may have acquired this TA system from neighboring bacteria within biological niches since toxin genes can be carried on plasmids, bacteriophages or within bacterial chromosomes. In addition, Bryant et al. recently described models for Mab evolution within human hosts upon sustained infection^51^. By whatever means, acquisition of TA systems is thought to endow bacteria with a competitive edge in the battle for survival under harsh conditions where competition for scarce nutrients is great and stress is frequent. For example, the *M. tuberculosis* VapC4 toxin upregulates pathways required for protection against oxidative and copper stresses imparted by macrophages during infection^27^. In contrast to the ~90 TA systems harbored by the pathogenic H37Rv *M. tuberculosis* strain, most of the 128 clinical Mab strains we identified carry only one TA system. VapBC5 was present in ~25% of these 128 strains, and the only apparent TA system in those clinical isolates. However, at least in the case of VapC5, this single TA system has a very potent effect that is predicted to contribute to the tenacity of Mab after long and aggressive treatment regimens (illustrated in Fig. 9). This multifaceted yet circumscribed reprogramming of cell physiology exclusively toward antibiotic resistance, the most prominent feature of Mab, has not been shown for any TA system to date. The fact that Mab strains typically acquire only one TA system may reflect their emergence as pathogens as the antibiotic age began. If antibiotic exposure were its first barrier to survival, the selective pressure to override its killing action could have favored acquisition of a TA system followed by its functional adaptation to enable codon-specific physiological reprogramming such as that documented in this work. Given the potent advantage that acquisition of VapBC5 has in maintaining viability in human infections, it is surprising that it is not present in more clinical strains. However, Mab is generally a free-living environmental saprophyte predominantly found in water and soil that evolved into a human pathogen. This evolutionary path may have limited opportunities for VapBC5 TA module acquisition, so the penetrance of advantageous traits may simply take more time for this relatively young pathogen. The ancient *M. tuberculosis* pathogen has ~90 TA systems that were acquired and functionally fine-tuned toward the specific needs of this pathogen during its projected 73,000-year existence^52^.

As with nearly all bacterial type II TA systems, and all in mycobacteria, the precise environmental triggers that activate VapC5 through selective reduction in cognate antitoxin levels remains unknown. However, here we used low level VapC5 expression to mimic natural toxin activation in a strain lacking a VapBC5 TA system. First and foremost, VapC5 expression results in a hallmark phenotype of Type II TA systems, cell growth arrest (Fig. 1D). As with the *M. tuberculosis* VapC4 toxin^27^, VapC5-expressing cells still manage to adroitly reprogram Mab physiology at the transcriptional and translational level despite their growth arrested state.

The inactivation of the tRNA servicing the most abundant Ser UCG codon in Mab along with a fraction of tRNA^fMet^ is expected to lead to widespread ribosome stalling. The striking upregulation of *whiB7* by VapC5 appears to be linked to this widespread ribosome stalling since *whiB7* is regulated by uORF-mediated transcription attenuation^42^ and is thought to sense ribosome stalling^53^. Ribosome availability is known to control bacterial growth rate and bacteria cannot survive below a threshold level of ribosomes^54^. Thus, the extensive ribosome stalling after VapC5 cleavage of these tRNAs would likely activate signals to overproduce ribosomes as cells remain growth arrested (which provides survival advantages in the presence of many antibiotics) and simply try to stay alive. But this effect on ribosome synthesis permeates into the realm of antibiotic efficacy as well. The overproduction of ribosomes is a clever means—essentially via high copy suppression—to ensure that cells have enough functional ribosomes while the 23 S rRNA inactivating Erm(41) methylase and other WhiB7-activated enzymes are simultaneously inactivating them. The overproduction of ribosomes should also abrogate the efficacy of amikacin and any other ribosome-targeting antibiotic used for treatment of Mab infections as depicted in Fig. 9. In fact, Mab and *Mycobacterium avium* mutations in the 16 S rRNA region targeted by amikacin within the ribosome active center leads to amikacin resistance^55^. In addition, the stockpiling of translational capacity—VapC5 also enhances translation factor synthesis along with ribosomes (Fig. 4E)—has been previously shown to support rapid recovery of stressed *Escherichia coli* cells^56^. Therefore, this toxin-mediated phenotypic shift is predicted to disrupt the efficacy of all first line Mab antibiotics (which target ribosomes or cell wall synthesis) as well as support robust recovery when the antibiotic is removed (illustrated in Fig. 9).

Consistent with this prediction, VapC5-expressing Mab cells supported an increase in the recovery of persisters against the two classes of first line antibiotics amikacin and cefoxitin as well as the newer antibiotic tedizolid. These results indicate that the killing action of amikacin, cefoxitin, and tedizolid is weakened when VapC5 is expressed, leading to a larger pool of persisters that can seed growth once the antibiotic is removed (Figs. 8, 9). Note that this effect is over and above the already well documented intrinsic resistance exhibited by Mab ATCC 19977 cells exposed to antibiotics^40,44,57^. However, while the mechanism enlisted by VapC5 shares some molecular features when Mab ATCC 19977 is treated with antibiotics (i.e., WhiB7 and erm(41) activation), Fig. 3A illustrates clear differences in the pattern of transcriptional reprogramming by VapC5 versus antibiotic exposure. For example, VapC5 induces expression of several other proposed WhiB family members^41^, suggesting that future therapeutic approaches designed to exclusively target WhiB7 inactivation may not be effective for the significant portion of clinical strains harboring VapC5. None of the seven other WhiB family members were represented in RNA-seq datasets when Mab ATCC 19977 cells were treated with erythromycin or kanamycin^44^. Also, Mab ATCC 19977 cells treated with amikacin upregulate *whiB7*, but not *eis2* or ribosomal protein transcripts^40^. Therefore, upregulation of ribosomal protein transcripts and newly synthesized ribosomal proteins appears to be unique to VapC5.

In summary, VapC5 expression does not completely cripple the cell but instead restrains growth while launching a counterattack that potentiates the most vexing trait of this pathogen—cell survival upon antibiotic stress. The acquisition of a single VapBC5 TA system in many Mab clinical strains represents an adaptation that puts ribosome biosynthesis in overdrive even though cells are in a growth arrested state. These two phenotypes are predicted to override the efficacy of the most recommended treatment regimen for Mab infections: clarithromycin with amikacin plus cefoxitin or imipenem^11^ (Fig. 8). Clarithromycin and amikacin both target the ribosome while the killing action of cefoxitin and imipenem requires actively dividing cells (VapC5-expressing cells are not; Fig. 1D) because they target cell wall synthesis. Armed with a better understanding of the molecular underpinnings of the role of VapC5 in persistence, simple PCR screening of clinical isolates for the presence of the VapBC5 TA system should guide the course of treatment toward other antibiotics with cellular targets that are not predicted to be affected by VapC5^21^.

## Methods

### Toxin-antitoxin identification

Mab VapC5 toxin (accession number WP_074293606.1) was identified  by BLASTP against all *M. tuberculosis* VapC toxins and so named for its highest similarity to *M. tuberculosis* VapC5 (Rv0627). For VapC5 to be considered a toxin within a bone fide TA system, an out-of-frame upstream putative antitoxin gene must be present. A putative *vapB5* (accession number WP_074293607.1) was identified upstream of each of the 32 Mab clinical strains harboring a *vapC5* toxin gene.

### Strains, plasmids and reagents

All experiments were performed using *Mycobacteroides abscessus* strain L948 (ATCC 19977). Mab L948 cells were grown in 7H9 Middlebrook medium supplemented to a final concentration of 0.05% Tween 80, 0.5% bovine albumin, 0.2% dextrose, 0.085% NaCl, 0.1% casamino acids and 25 μg/ml kanamycin or zeocin a 25 μg/ml (for plasmid selection). All cultures were grown at 37 °C under constant agitation at 170 revolutions per minute (rpm). VapC5 was synthesized with the addition of 5’ NdeI and 3’ HindIII restriction sites and provided as an NdeI/HindIII insert in plasmid pUC57 (GenScript). The *vapC5* gene was digested from pUC57 and cloned into the NdeI and HindIII sites adjacent to an anhydrotetracycline (ATc) inducible promoter in the pMC1s plasmid^58^. VapC5 expression was induced by adding ATc 200 ng/ml (Clontech) to the media when cells reached an OD_600_ of ~0.1 and compared to an empty plasmid control with ATc added at an OD_600_ of ~0.1 as well. For co-expression experiments, VapB5 antitoxin was synthesized with the addition of 5’ EcoRI and 3’ NdeI restriction sites and provided as an EcoRI/NdeI insert in plasmid pUC57 (GenScript). The *vapB5* gene was cloned into the EcoRI and NdeI sites of the isovaleronitrile (IVN) inducible plasmid pNIT and transformed into Mab competent cells already containing pMC1s-*vapC5*. For *vapB5* and *vapC5* co-expression, cells were induced by adding ATc and IVN to a final concentration of 100 ng/ml and 0.5 μM, respectively.

### RNA isolation

Total RNA was extracted from ~200 ml of Mab cells containing either *vapC5*-pMC1s or empty pMC1s grown for 6 hrs with the addition of 200 ng/mL ATc. Cells were centrifuged at 2300 × *g* at 4 °C for 10 min, cell pellets resuspended in 1 ml of Tri reagent (Zymo Research) and transferred to 2 ml lysing kits tubes (Bertin Corp.) containing 0.1 mm glass beads. Cells were lysed with a Precellys Evolution homogenizer (Bertin Corp.) using four 30 s cycles with the agitation set to 9000 rpm interspersed with 2 min cooling periods. The lysate was centrifuged at 16,000 × *g* for 5 min at 4 °C and RNA was isolated from the supernatant using the Direct-zol RNA Miniprep Plus extraction kit (Zymo Research). Isolated RNA was subsequently treated with 1 U of Turbo DNase for 30 min at 37 °C and purified and eluted in 45 μl of RNase-free water using the RNA Clean and Concentrator kit (Zymo Research). The RNA concentration was measured using a BioSpectrometer (Eppendorf) with a μCuvette.

### Northern analysis of tRNA levels

To detect Mab tRNA, specific DNA oligonucleotides complementary to the 5’ or 3’ end of Mab tRNA^SerCGA^ or tRNA^fMet^ (NWO3166, 5’-TTCGAGGCGTGCTCCTTAGGC-3’ and NWO3111, 5’-CGAGCTGCTCCACCCCGC-3’, respectively) were radiolabeled at the 5′ end with T4 polynucleotide kinase (New England Biolabs) and [γ-^32^P]ATP (PerkinElmer) for 1 h at 37 °C. Total RNA (1 μg) from Mab was resolved on a 15% polyacrylamide, 7 M urea gel and stained with SYBR Gold (Invitrogen) to assess and ensure overall quality. The RNA was transferred to a nylon Hybond-N+ membrane (GE Healthcare) and hybridized with the ^32^P-labeled oligonucleotides overnight at ~10 °C below the oligonucleotide T_m_. The membranes were washed with 2 X SSC and 0.1% SDS at the temperature just below the oligonucleotide T_m_ for 15 min, followed by a secondary wash with 0.1 X SSC and 0.1% SDS at the same temperature for 15 min. The membranes were then exposed to phosphorimager screens for 2 h and imaged with the Typhoon FLA 9500 system (GE Healthcare).

### Labeling of newly synthesized Mab proteins

To assess newly synthesized protein levels following VapC5 induction, cells were grown to an OD of ~0.1 and divided into induced and uninduced samples. After ATc addition for 6, 8, or 12 h, cells were collected by centrifugation and resuspended in new media without casamino acids. Cells were then incubated at 37 °C for 30 min followed by addition 50 μM azidohomoalanine (AHA, AnaSpec) for 2 h. Cells were pelleted by centrifugation at 4 °C and washed with 1X PBST (137 mM NaCl, 2.7 mM KCl, 10 mM Na_2_HPO_4_, 1.8 mM KH_2_PO_4,_ and 0.05% Tween 80). Cell pellets were resuspended in 2% CHAPS, 8 M Urea buffer and were lysed with a Precellys Evolution homogenizer using three cycles of 30 s at 9000 rpm, interspersed with 2 min cooling periods. The lysates were pelleted at 16,000 × *g* at 4 °C for 10 min, and the AHA-labeled proteins in the supernatant were selectively captured using alkyne-coated agarose beads from the Click-iT Protein Enrichment Kit (Thermo Fisher) following the manufacturer’s protocol.

### 5’ RNA-seq

Construction of 5’-dependent libraries was performed as previously described^38^. The amplified library fragments of 150–450 bp were gel purified from a 10% TBE PAGE gel and sequenced using a HiSeq 2500 platform or similar. The resulting FASTQ files had the adapter sequences and the first 6 nucleotides of the 5’ end trimmed using Trimmomatic 0.36.5^59^. Reads were then trimmed to 20 nts using Trim Galore 0.6.7 and reads containing fewer than 20 nucleotides were excluded. The remaining reads were mapped to the Mab genome (NCBI accession: CU458896.1 with the addition of the two putative tRNAs not annotated in the Mab ATCC 19977 genome that were identified in the GtRNAdb tRNA database^60^) using Bowtie 1.2 applying the parameters -n 0 -l 20^61^. Read counts were normalized to sequencing depth and expressed as reads per million of mapped reads. Fold change was calculated using the ratio for each position by dividing the counts in induced sample/counts in uninduced sample. Positions that had 0 counts in the uninduced library were adjusted to a pseudo-count of 1. Reads were only considered if they had a fold change ≥60. The *k*pLogo program^62^ was utilized to visualize nucleotide frequency of the top hits using the default parameters except the k-mer length was set to 3 and residues were scaled according to Bonferroni corrected *p* value. Amino acid frequency of the top hits was also visualized using *k*pLogo^62^ using the following parameters: -seq 1 -weight 2 -alphabet protein -max_k 2 -shift 0 -startPos 1 -minCount 0.01 -pseudo 1 -region 1,0 -plot b -pc 0.01 -stack_order 1 -fix 0.75.

### RNA-seq

In order to deplete 16 S/23 S ribosomal RNA from extracted RNA, samples were subjected to the rRNA depletion method described in ref. ^63^. In short, extracted RNA was incubated with biotinylated oligonucleotides designed to specifically hybridize to the 23 S, 16 S and 5 S rRNA of a variety of bacterial species. Biotinylated rRNA was then bound to magnetic streptavidin beads, leading to the depletion of ~75% of rRNA. Approximately 100 ng of rRNA-depleted RNA was used to generate the libraries utilizing using the NEBNext Ultra II Directional RNA Library Prep Kit for Illumina (New England Biolabs) and sequenced on an Illumina HiSeq 4000. The resulting sequences were mapped to the Mab reference genome (NCBI accession: CU458896.1) using the default parameters of Bowtie 1.2. Stringtie 1.3.439 and LimmaVoom 3.83.3 programs were used for transcript assembly and differential expression analysis, respectively.

### Proteomics

To assess newly synthesized protein in Mab cultures ±VapC5 quadruplicate samples were labeled with AHA for 2 hrs as described previously. We selectively captured newly synthesized proteins using an alkyne-containing column from the Click-iT™ Protein Enrichment Kit (ThermoFisher) followed by in-column trypsin digestion. Digests were analyzed in two separate runs and combined. Data were analyzed as previously described^38^. Data are presented as estimated log_2_ ratios of +Vap5/-VapC5 samples. Q-values are calculated using the fdrtool package of Strimmer^64^ with *q* ≤ 0.05 deemed significant.

### cDNA synthesis and qPCR

cDNA synthesis was performed on RNA using SuperScript™IV Reverse Transcriptase (Invitrogen) and 50 µM random hexamers following the manufacturer’s protocol. The real-time quantitative PCR (qPCR) was performed using the QuantStudio™ 3 System (ThermoFisher). As recommended, all amplifications were carried out with Platinum® SYBR® Green qPCR SuperMix-UDG with ROX using 2.5 µL of the final cDNA derived from 200 ng of total RNA. Reactions were prepared according to the manufacturer’s protocol. PCR conditions were 95 °C for 10 min followed by 40 cycles of 95 °C for 15 s and 60 °C for 60 s. The specificity of each pair of primers was checked by melting curve analysis (95 °C for 15 s, 60 °C for 1 min and a continuous raise in temperature to 95 °C at 0.3 °C/s ramp rate followed by 95 °C for 15 s). To check for reproducibility, three technical and three biological replicates were used. The following oligonucleotides were used: *sigA* (NWO3525 5’-CACATGGTCGAGGTCATCAA-3’ and NWO3526 5’-TGGATTTCCAGCACCTTCTC-3’), *vapC5* (NWO3527 5’-CGGAATTGCACTTTGGTGTC-3’ and NWO3528 5’-CGATCGTTGAAGCAGGAGTAG-3’), *whiB7* (NWO3515 5’-CCTGTGGTTCGCGGAAA-3’ and NWO3516 5’-CCCTGCTCAAGAATCTCACC-3’), *erm(41)* (NWO3517 5’- GCACTGATACGGAGTCTCTTG-3’ and NWO3518 5’-CTCGCTTCGCATGTTTGTG-3’), *eis2* (NWO3519 5’-GAGCTTCATGTGCAAGAGGT-3’ and NWO3520 5’-GCGCCGTGATACTTGATCTT-3’), *aac(2’)* (NWO3521 5’-TCTGGTACCACGGCATACT-3’ and NWO3522 5’-CGCAACGCCTTCCACATA-3’), *tap* (NWO3523 5’-ACTGCCCTGGCTTGTATTG-3’ and NWO3524 5’-CCGAGATGAGTGTCGAGAAGA-3’). Calculation of the normalized gene expression (relative to the *sigA* housekeeping gene) were obtained based on ∆∆CT.

### Antibiotic MICs

Stock solutions of 50 mg/mL amikacin and cefoxitin (Sigma) in sterile reverse osmosis water were prepared fresh on the day of the experiment. A 10 mg/ml stock of tedizolid (MedChemExpress USA) in dimethyl sulfoxide was prepared, aliquoted, and stored at −80 °C. ATCC 19977 Mab cells were inoculated in 7H9 media (supplemented as above) until they reached an OD_600_ = 0.1 (1 × 10^7^ CFU/ml). The cells were diluted to 1 × 10^5^ cells per 100 µl. Antibiotics were diluted to desired concentrations (0–128 µg/ml amikacin, 0–256 µg/ml cefoxitin, and 0.01–250 µg/mL tedizolid) and 100 µl of cells were added to applicable wells in a 96 well plate. The plates were incubated as recommended for each antibiotic at 37 °C and the MIC was visually determined as antibiotic concentration of the first clear well in the dilution series^65^.

### Persister cell assay

Mab cells containing either empty vector pMC1s (-VapC5) or *vapC5-*pMC1s (+VapC5) were grown at 37 °C until they reached an OD_600_ ~ 0.1 or diluted from cultures with an OD_600_ ≤ 0.25. 1 ml of VapC5 induced cultures and empty vector controls were aliquoted, serially diluted and plated for CFU/ml. 160 µg/ml of amikacin (10 X MIC), 320 µg/ml of cefoxitin (10 X MIC), or 15 µg/ml of tedizolid (5 X MIC), was added to each toxic VapC5 culture and the empty vector controls. After a period of antibiotic exposure, 1 ml of treated cells were pelleted at 15,000 × *g* for 2 min. The cells were washed two times with 1X PBST, serially diluted and plated to determine CFU/ml. The antibiotic exposure timepoints were compared to the pre-treatment timepoint to determine cell survival rate.

The following guidelines must be followed for consistent results: (1) -VapC5 control cells must reach MDK_99_ within 24 h for amikacin, within 12 h for cefoxitin, and within 7 days for tedizolid; (2) +VapC5 cells must be freshly transformed ~1 week prior to each experiment; (3) +VapC5 cells must display toxicity after 5–6 h of ATc induction; (4) samples displaying clear antibiotic resistance must be eliminated; (5) samples from +VapC5 cells exhibiting clear outlier behavior with time relative to other replicates should be removed from data analysis; (6) cells that are diluted to ~0.1 before addition of ATc should not originate from cultures with OD_600_ readings exceeding 0.25; (7) perform initial CFU/ml counts ~4–5 days following plating, then recount ~10–14 days after initial plating (which typically result in 10–30% more colonies above the initial plate count) (8) compare plate counts of -VapC5 to +VapC5 cells from matched time of ATc exposure and matched incubation times after plating, usually the 10–14 day plates are most representative; (9) plates containing 20–300 colonies within a dilution series are considered significant (10) colony counts within a dilution series should be representative of the dilution pattern to ensure cell clumping is not skewing the data.

### Statistical analyses

Statistical analyses for growth profiles and bar graphs were performed using GraphPad Prism software 9.5.1.

### Reporting summary

Further information on research design is available in the Nature Portfolio Reporting Summary linked to this article.

## Supplementary information

Supplementary Information

Description of Additional Supplementary Files

Supplementary Data 1

Supplementary Data 2

Supplementary Data 3

Supplementary Data 4

Supplementary Data 5

Supplementary Data 6

Supplementary Data 7

Reporting Summary

## Data Availability

The RNA sequencing data generated in this study have been deposited in the NCBI Sequence Read Archive under accession number PRJNA942981. The mass spectrometry proteomics data generated in this study have been deposited to the ProteomeXchange Consortium via the PRIDE partner repository with the dataset identifier PXD025047. The supplementary data generated in this study are provided as Supplementary Data 1–7, Supplementary Fig. 1 and a single Source File. Source data are provided with this paper.

## Source data

Source Data

## References

[1] Benwill JL, Wallace RJ (2014). Mycobacterium abscessus: challenges in diagnosis and treatment. Curr. Opin. Infect. Dis..

[2] Brown-Elliott BA, Nash KA, Wallace RJ (2012). Antimicrobial susceptibility testing, drug resistance mechanisms, and therapy of infections with nontuberculous mycobacteria. Clin. Microbiol. Rev..

[3] Johansen MD, Herrmann JL, Kremer L (2020). Non-tuberculous mycobacteria and the rise of Mycobacterium abscessus. Nat. Rev. Microbiol..

[4] Mougari F (2017). Selection of resistance to clarithromycin in mycobacterium abscessus subspecies. Antimicrob. Agents Chemother..

[5] Nessar R, Cambau E, Reyrat JM, Murray A, Gicquel B (2012). Mycobacterium abscessus: a new antibiotic nightmare. J. Antimicrob. Chemother..

[6] Novosad SA (2016). Treatment of Mycobacterium abscessus Infection. Emerg. Infect. Dis..

[7] Park J, Cho J, Lee CH, Han SK, Yim JJ (2017). Progression and treatment outcomes of lung disease caused by mycobacterium abscessus and mycobacterium massiliense. Clin. Infect. Dis. Off. Publ. Infect. Dis. Soc. Am..

[8] Johnson MM, Odell JA (2014). Nontuberculous mycobacterial pulmonary infections. J. Thorac. Dis..

[9] Lee MR (2015). Mycobacterium abscessus complex infections in humans. Emerg. Infect. Dis..

[10] Medjahed H, Gaillard JL, Reyrat JM (2010). Mycobacterium abscessus: a new player in the mycobacterial field. Trends Microbiol..

[11] Mougari F (2016). Infections caused by Mycobacterium abscessus: epidemiology, diagnostic tools and treatment. Expert Rev. Anti Infect. Ther..

[12] Bryant JM (2013). Whole-genome sequencing to identify transmission of Mycobacterium abscessus between patients with cystic fibrosis: a retrospective cohort study. Lancet.

[13] Bryant JM (2016). Emergence and spread of a human-transmissible multidrug-resistant nontuberculous mycobacterium. Science.

[14] Doyle RM (2020). Cross-transmission is not the source of new mycobacterium abscessus infections in a multicenter cohort of cystic fibrosis patients. Clin. Infect. Dis.: Off. Publ. Infect. Dis. Soc. Am..

[15] Bronson RA (2021). Global phylogenomic analyses of Mycobacterium abscessus provide context for non cystic fibrosis infections and the evolution of antibiotic resistance. Nat. Commun..

[16] Strathdee SA, Hatfull GF, Mutalik VK, Schooley RT (2023). Phage therapy: from biological mechanisms to future directions. Cell.

[17] Dedrick RM (2021). Potent antibody-mediated neutralization limits bacteriophage treatment of a pulmonary Mycobacterium abscessus infection. Nat. Med..

[18] Chen J (2019). Clinical efficacy and adverse effects of antibiotics used to treat mycobacterium abscessus pulmonary disease. Front. Microbiol..

[19] Diel R (2017). Microbiological and clinical outcomes of treating non-mycobacterium avium complex nontuberculous mycobacterial pulmonary disease: a systematic review and meta-analysis. Chest.

[20] Martiniano SL, Sontag MK, Daley CL, Nick JA, Sagel SD (2014). Clinical significance of a first positive nontuberculous mycobacteria culture in cystic fibrosis. Ann. Am. Thorac. Soc..

[21] Wu ML, Aziz DB, Dartois V, Dick T (2018). NTM drug discovery: status, gaps and the way forward. Drug Discov. Today.

[22] Harms A, Maisonneuve E, Gerdes K (2016). Mechanisms of bacterial persistence during stress and antibiotic exposure. Science.

[23] Moldoveanu AL, Rycroft JA, Helaine S (2021). Impact of bacterial persisters on their host. Curr. Opin. Microbiol..

[24] Ronneau S, Helaine S (2019). Clarifying the link between toxin-antitoxin modules and bacterial persistence. J. Mol. Biol..

[25] Fraikin N, Goormaghtigh F, Van Melderen L (2020). Type II toxin-antitoxin systems: evolution and revolutions. J. Bacteriol..

[26] Sala A, Bordes P, Genevaux P (2014). Multiple toxin-antitoxin systems in Mycobacterium tuberculosis. Toxins.

[27] Barth VC (2021). Mycobacterium tuberculosis VapC4 toxin engages small ORFs to initiate an integrated oxidative and copper stress response. Proc. Natl Acad. Sci. USA.

[28] Barth VC, Woychik NA (2019). The sole mycobacterium smegmatis MazF toxin targets tRNA(Lys) to impart highly selective, codon-dependent proteome reprogramming. Front. Genet..

[29] Chauhan U, Barth VC, Woychik NA (2022). tRNA(fMet) inactivating mycobacterium tuberculosis VapBC toxin-antitoxin systems as therapeutic targets. Antimicrob. Agents Chemother..

[30] Cintrón M (2019). Accurate target identification for Mycobacterium tuberculosis endoribonuclease toxins requires expression in their native host. Sci. Rep..

[31] Cruz JW (2015). Growth-regulating Mycobacterium tuberculosis VapC-mt4 toxin is an isoacceptor-specific tRNase. Nat. Commun..

[32] Walling LR, Butler JS (2018). Homologous VapC toxins inhibit translation and cell growth by sequence-specific cleavage of tRNA(fMet). J. Bacteriol..

[33] Winther K, Tree JJ, Tollervey D, Gerdes K (2016). VapCs of Mycobacterium tuberculosis cleave RNAs essential for translation. Nucleic Acids Res.

[34] Winther KS, Gerdes K (2011). Enteric virulence associated protein VapC inhibits translation by cleavage of initiator tRNA. Proc. Natl Acad. Sci. USA.

[35] Cruz JW, Woychik NA (2016). tRNAs taking charge. Pathog. Dis..

[36] Schifano JM (2014). An RNA-seq method for defining endoribonuclease cleavage specificity identifies dual rRNA substrates for toxin MazF-mt3. Nat. Commun..

[37] Schifano JM (2016). tRNA is a new target for cleavage by a MazF toxin. Nucleic Acids Res..

[38] Barth VC (2019). Toxin-mediated ribosome stalling reprograms the Mycobacterium tuberculosis proteome. Nat. Commun..

[39] McClain WH, Schneider J, Bhattacharya S, Gabriel K (1998). The importance of tRNA backbone-mediated interactions with synthetase for aminoacylation. Proc. Natl Acad. Sci. USA.

[40] Hurst-Hess K, Rudra P, Ghosh P (2017). Mycobacterium abscessus WhiB7 Regulates a Species-Specific Repertoire of Genes To Confer Extreme Antibiotic Resistance. Antimicrob. Agents Chemother..

[41] Pryjma M, Burian J, Kuchinski K, Thompson CJ (2017). Antagonism between front-line antibiotics clarithromycin and amikacin in the treatment of mycobacterium abscessus infections is mediated by the whiB7 gene. Antimicrob. Agents Chemother..

[42] Lee JH, Lee EJ, Roe JH (2022). uORF-mediated riboregulation controls transcription of whiB7/wblC antibiotic resistance gene. Mol. Microbiol..

[43] Nash KA, Brown-Elliott BA, Wallace RJ (2009). A novel gene, erm(41), confers inducible macrolide resistance to clinical isolates of Mycobacterium abscessus but is absent from Mycobacterium chelonae. Antimicrob. Agents Chemother..

[44] Miranda-CasoLuengo AA, Staunton PM, Dinan AM, Lohan AJ, Loftus BJ (2016). Functional characterization of the Mycobacterium abscessus genome coupled with condition specific transcriptomics reveals conserved molecular strategies for host adaptation and persistence. BMC Genom..

[45] Huang da W, Sherman BT, Lempicki RA (2009). Systematic and integrative analysis of large gene lists using DAVID bioinformatics resources. Nat. Protoc..

[46] Huang da W, Sherman BT, Lempicki RA (2009). Bioinformatics enrichment tools: paths toward the comprehensive functional analysis of large gene lists. Nucleic Acids Res..

[47] Shajani Z, Sykes MT, Williamson JR (2011). Assembly of bacterial ribosomes. Annu Rev. Biochem..

[48] Deuerling E, Gamerdinger M, Kreft SG (2019). Chaperone interactions at the ribosome. Cold Spring Harb. Perspect. Biol..

[49] Daley CL (2020). Treatment of nontuberculous mycobacterial pulmonary disease: an official ATS/ERS/ESCMID/IDSA clinical practice guideline: executive summary. Clin. Infect. Dis. Off. Publ. Infect. Dis. Soc. Am..

[50] Balaban NQ (2019). Definitions and guidelines for research on antibiotic persistence. Nat. Rev. Microbiol..

[51] Bryant JM (2021). Stepwise pathogenic evolution of Mycobacterium abscessus. Science.

[52] Cardona PJ, Catala M, Prats C (2020). Origin of tuberculosis in the Paleolithic predicts unprecedented population growth and female resistance. Sci. Rep..

[53] Poulton NC, Rock JM (2022). Unraveling the mechanisms of intrinsic drug resistance in Mycobacterium tuberculosis. Front. Cell Infect. Microbiol..

[54] Dai X, Zhu M (2020). Coupling of ribosome synthesis and translational capacity with cell growth. Trends Biochem. Sci..

[55] Kim SY (2021). Association between 16S rRNA gene mutations and susceptibility to amikacin in Mycobacterium avium Complex and Mycobacterium abscessus clinical isolates. Sci. Rep..

[56] Mori M, Schink S, Erickson DW, Gerland U, Hwa T (2017). Quantifying the benefit of a proteome reserve in fluctuating environments. Nat. Commun..

[57] Yam YK, Alvarez N, Go ML, Dick T (2020). Extreme drug tolerance of Mycobacterium abscessus “Persisters”. Front. Microbiol..

[58] Ehrt S (2005). Controlling gene expression in mycobacteria with anhydrotetracycline and Tet repressor. Nucleic Acids Res..

[59] Bolger AM, Lohse M, Usadel B (2014). Trimmomatic: a flexible trimmer for Illumina sequence data. Bioinformatics.

[60] Chan PP, Lowe TM (2016). GtRNAdb 2.0: an expanded database of transfer RNA genes identified in complete and draft genomes. Nucleic Acids Res..

[61] Langmead B, Trapnell C, Pop M, Salzberg SL (2009). Ultrafast and memory-efficient alignment of short DNA sequences to the human genome. Genome Biol..

[62] Wu X, Bartel DP (2017). kpLogo: positional k-mer analysis reveals hidden specificity in biological sequences. Nucleic Acids Res..

[63] Culviner P, Guegler C, Laub M (2020). A simple, cost-effective, and robust method for rRNA depletion in RNA-sequencing studies. mBio.

[64] Strimmer K (2008). fdrtool: a versatile R package for estimating local and tail area-based false discovery rates. Bioinformatics.

[65] Schon T (2020). Antimicrobial susceptibility testing of Mycobacterium tuberculosis complex isolates - the EUCAST broth microdilution reference method for MIC determination. Clin. Microbiol. Infect. Off. Publ. Eur. Soc. Clin. Microbiol. Infect. Dis..

