# Supplementary material for: Mycobacterium abscessus VapC5 toxin potentiates evasion of antibiotic killing by ribosome overproduction and activation of multiple resistance pathways
Source: Nat Commun. 2023 Jun 22;14:3705. doi: 10.1038/s41467-023-38844-4 (PMC10287673; doi:10.1038/s41467-023-38844-4)
Supplement: Supplementary file 1 — Supplementary Information [file 41467_2023_38844_MOESM1_ESM.pdf]

SUPPLEMENTARY FIGURE 1

A

UGGCGUGUCCGAGCGGCCUAAGGAGCACGCCUCGAAAGCGUGUGACGGGUAACCCCCGUCCGAGGGUUCA tRNA$^{Ser\text{-}CGA}$

CGGGGUGGAGCAGCUCGGUAGCUCGCUGGGCUCAUAACCCAGAGGUCGCAGGUUCAAAUCCUGUCCCCGC tRNA^{fMet}

B

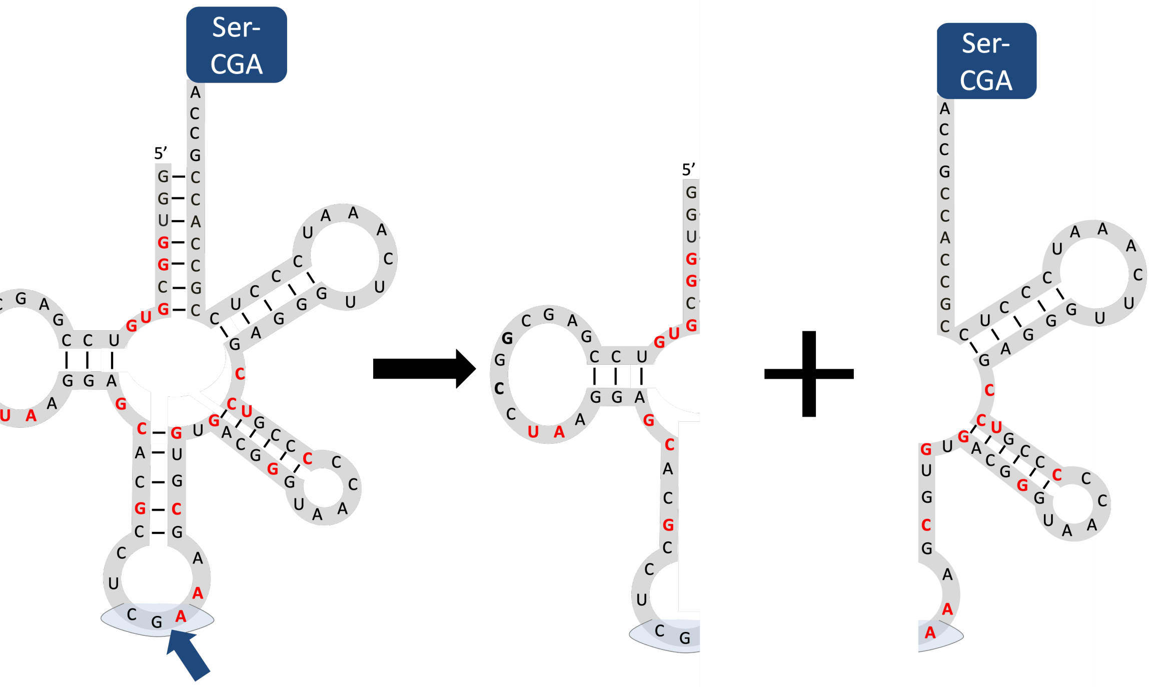

Supplementary Fig. 1. VapC5 cleavage is sequence and structure specific. A. Alignment of the two VapC5 tRNA targets; anticodon sequences underlined. B. VapC5 tRNA^{SerCGA} diagram demonstrating location of nt identities (red) based on alignment in A. Blue arrow denotes VapC5 cleavage site.
