# Supplementary material for: Mycobacterium abscessus VapC5 toxin potentiates evasion of antibiotic killing by ribosome overproduction and activation of multiple resistance pathways
Source: Nat Commun. 2023 Jun 22;14:3705. doi: 10.1038/s41467-023-38844-4 (PMC10287673; doi:10.1038/s41467-023-38844-4)
Supplement: Supplementary file 2 — Description of Additional Supplementary Files [file 41467_2023_38844_MOESM2_ESM.pdf]

Description of Additional Supplementary Files

File Name: Supplementary Data 1
Description: 5’ RNA-seq mRNA dataset for ATCC 19977 *M. abscessus* cells ±VapC5 6 hours following ATc addition. Cleavage is observed immediately before the capitalized nucleotide. Flanking DNA sequences were added 25 nt up- and downstream of the cleavage site.

File Name: Supplementary Data 2
Description: RNA-seq dataset for ATCC 19977 *M. abscessus* cells ±VapC5 6 hours following ATc addition.

File Name: Supplementary Data 3
Description: DAVID (**D**atabase for **A**nnotation, **V**isualization and **I**ntegrated **D**iscovery) analysis of the RNA-seq data from Supplementary Data 2. All upregulated categories are compiled in the ‘upregulated’ tab, while downregulated categories are compiled in the ‘downregulated’ tab.

File Name: Supplementary Data 4
Description: Mass spectrometry data of the newly synthesized proteins in ATCC 19977 *M. abscessus* ±VapC5. Tab ‘8h’ refers to data obtained from cells after 8 hours of induction; tab ‘10h’ refers to data obtained from cells after 10 hours of induction; tab ‘14h’ refers to data obtained from cells after 14 hours of induction.

File Name: Supplementary Data 5
Description: DAVID (**D**atabase for **A**nnotation, **V**isualization and **I**ntegrated **D**iscovery) analysis of the mass spectrometry data of newly synthesized proteins in ATCC 19977 *M. abscessus* ±VapC5 cells after 8, 10 or 14 hours of induction from Supplementary Data 5. Each time point has a respective upregulated or downregulated tab.

File Name: Supplementary Data 6
Description: 5’ RNA-seq mRNA dataset of MC^2-155 *M. smegmatis* ±VapC5 6 hours following ATc addition. Cleavage is observed immediately before the capitalized nt. Flanking DNA sequences were added 25 nt up- and downstream of the cleavage site.

File Name: Supplementary Data 7
Description: Persister assay data from ATCC 19977 *M. abscessus* cells ±VapC5. Amikacin data, ‘AMK’ tab; cefoxitin data, ‘FOX’ tab; tedizolid data, ‘TED’ tab.
