# Supplementary material for: Mycobacterium abscessus VapC5 toxin potentiates evasion of antibiotic killing by ribosome overproduction and activation of multiple resistance pathways
Source: Nat Commun. 2023 Jun 22;14:3705. doi: 10.1038/s41467-023-38844-4 (PMC10287673; doi:10.1038/s41467-023-38844-4)
Supplement: Supplementary file 10 — Reporting Summary [file 41467_2023_38844_MOESM10_ESM.pdf]

nature portfolio

Corresponding author(s): Nancy Woychik

Last updated by author(s): May 11, 2023

Reporting Summary

Nature Portfolio wishes to improve the reproducibility of the work that we publish. This form provides structure for consistency and transparency in reporting. For further information on Nature Portfolio policies, see our Editorial Policies and the Editorial Policy Checklist.

Statistics

For all statistical analyses, confirm that the following items are present in the figure legend, table legend, main text, or Methods section.

n/a	Confirmed	
☐	☒	The exact sample size (*n*) for each experimental group/condition, given as a discrete number and unit of measurement
☐	☒	A statement on whether measurements were taken from distinct samples or whether the same sample was measured repeatedly
☐	☒	The statistical test(s) used AND whether they are one- or two-sided<br>*Only common tests should be described solely by name; describe more complex techniques in the Methods section.*
☒	☐	A description of all covariates tested
☐	☒	A description of any assumptions or corrections, such as tests of normality and adjustment for multiple comparisons
☐	☒	A full description of the statistical parameters including central tendency (e.g. means) or other basic estimates (e.g. regression coefficient) AND variation (e.g. standard deviation) or associated estimates of uncertainty (e.g. confidence intervals)
☐	☒	For null hypothesis testing, the test statistic (e.g. *F*, *t*, *r*) with confidence intervals, effect sizes, degrees of freedom and *P* value noted<br>*Give P values as exact values whenever suitable.*
☒	☐	For Bayesian analysis, information on the choice of priors and Markov chain Monte Carlo settings
☒	☐	For hierarchical and complex designs, identification of the appropriate level for tests and full reporting of outcomes
☒	☐	Estimates of effect sizes (e.g. Cohen's *d*, Pearson's *r*), indicating how they were calculated

Our web collection on statistics for biologists contains articles on many of the points above.

Software and code

Policy information about availability of computer code

Data collection	No data collection software was used.
Data analysis	For data analysis, the following software was used: Trimmomatic 0.36.5, Trim Galore 0.6.7, Bowtie 1.2, LimmaVoom 3.38.3, Stringtie 1.3.4, kpLogo web version (2017) and Prism GraphPad 9.5.1.

For manuscripts utilizing custom algorithms or software that are central to the research but not yet described in published literature, software must be made available to editors and reviewers. We strongly encourage code deposition in a community repository (e.g. GitHub). See the Nature Portfolio guidelines for submitting code & software for further information.

Data

Policy information about availability of data

All manuscripts must include a data availability statement. This statement should provide the following information, where applicable:

- Accession codes, unique identifiers, or web links for publicly available datasets
- A description of any restrictions on data availability
- For clinical datasets or third party data, please ensure that the statement adheres to our policy

The RNA sequencing data generated in this study have been deposited in the NCBI Sequence Read Archive under accession number PRJNA942981 [https://dataview.ncbi.nlm.nih.gov/object/PRJNA942981?reviewer=2q8lqv45q2r310oahfrds1p745]. The mass spectrometry proteomics data generated in this study have been deposited to the ProteomeXchange Consortium via the PRIDE partner repository with the dataset identifier PXD025047 [https://proteomecentral.proteomexchange.org/cgi/GetDataset?ID=PXD025047]. The supplementary data generated in this study are provided as Supplementary Tables

1-7, Supplementary Data 1 and a single Source File.

Human research participants

Policy information about studies involving human research participants and Sex and Gender in Research.

Reporting on sex and gender	Not applicable
Population characteristics	Not applicable
Recruitment	Not applicable
Ethics oversight	Not applicable

Note that full information on the approval of the study protocol must also be provided in the manuscript.

Field-specific reporting

Please select the one below that is the best fit for your research. If you are not sure, read the appropriate sections before making your selection.

☒ Life sciences ☐ Behavioural & social sciences ☐ Ecological, evolutionary & environmental sciences

For a reference copy of the document with all sections, see nature.com/documents/nr-reporting-summary-flat.pdf

Life sciences study design

All studies must disclose on these points even when the disclosure is negative.

Sample size: For most experiments, we used triplicates which is widely accepted in the field of molecular biology.

Data exclusions: For conventional RNA-seq and proteomic analysis, reads were only considered if they reached a statistical value of $p \leq 0.05$. For 5' RNA-seq, reads were only considered if they had at least 50 counts (tRNA) with a fold change > 50. Specifically for the persister assay, the following guidelines must be followed for consistent results:
1) -VapC5 control cells must reach MDK99 within 24 hrs for amikacin, within 12 hrs for cefoxitin, and within 7 days for tedizolid;
2) +VapC5 cells must be freshly transformed ~1 week prior to each experiment;
3) +VapC5 cells must display toxicity after 5-6 hrs of ATc induction;
4) samples displaying clear antibiotic resistance must be eliminated;
5) samples from +VapC5 cells exhibiting clear outlier behavior with time relative to other replicates should be removed from data analysis;
6) cells that are diluted to ~0.1 before addition of ATc should not originate from cultures with OD600 reading exceeding 0.25;
7) perform initial cfu/ml counts ~4-5 days following plating, then recount ~10-14 days after initial plating (which typically results in 10-30% more colonies above the initial plate count)
8) compare plate counts of -VapC5 to +VapC5 cells from matched time of ATc exposure and matched incubation times after plating, usually the 10-14 day plates are most representative;
9) plates containing 30-300 colonies within a dilution series are considered significant
10) colony counts within dilution series should be representative of dilutions made to ensure cell clumping is not skewing the data.

Replication: We performed each experiment independently at least twice to ensure reproducibility of the reported results. All attempted replicates were successful.

Randomization: No randomization was required in our studies, as our study organisms are derived from well-established reference strains.

Blinding: Blinding was not necessary for data acquisition in our study, due to the nature of our research. Biological samples are assigned alias names during handling to avoid bias in treating certain samples differently. For data analysis, our datasets are treated as library names which are reassigned into their original sample description at the end of the workflow.

Reporting for specific materials, systems and methods

We require information from authors about some types of materials, experimental systems and methods used in many studies. Here, indicate whether each material, system or method listed is relevant to your study. If you are not sure if a list item applies to your research, read the appropriate section before selecting a response.

Materials & experimental systems

n/a	Involved in the study	
☒	☐	Antibodies
☒	☐	Eukaryotic cell lines
☒	☐	Palaeontology and archaeology
☒	☐	Animals and other organisms
☒	☐	Clinical data
☒	☐	Dual use research of concern

Methods

n/a	Involved in the study	
☒	☐	ChIP-seq
☒	☐	Flow cytometry
☒	☐	MRI-based neuroimaging
