# Supplementary material for: Mycobacterium abscessus VapC5 toxin potentiates evasion of antibiotic killing by ribosome overproduction and activation of multiple resistance pathways
Source: Nat Commun. 2023 Jun 22;14:3705. doi: 10.1038/s41467-023-38844-4 (PMC10287673; doi:10.1038/s41467-023-38844-4)
Supplement: Supplementary file 11 — Source Data [file 41467_2023_38844_MOESM11_ESM.pdf]

SOURCE DATA

Mycobacterium abscessus VapC5 toxin potentiates evasion of antibiotic killing by ribosome overproduction and activation of multiple resistance pathways

Eduardo A. Troian[1†], Heather M. Maldonado[1†], Unnati Chauhan[1], Valdir C. Barth[2] and Nancy A. Woychik[1,3]

	SigA	VapC5
Control 1	28.350	33.670
Control 2	28.349	33.667
Control 3	28.381	33.702
ATc 1	27.269	30.850
ATc 2	27.230	30.713
ATc 3	27.191	30.897
	SigA	VapC5
Control 1		5.320
Control 2		5.318
Control 3		5.320
ATc 1		3.581
ATc 2		3.482
ATc 3		3.706
	SigA	VapC5
ATc 1		-1.738
ATc 2		-1.835
ATc 3		-1.615
Fold change	SigA	VapC5
ATc 1		3.33634365388161
ATc 2		3.56881099186960
ATc 3		3.06217208199297
Log2 fold change		VapC5
ATc 1		1.738267899
ATc 2		1.835443497
ATc 3		1.614555359
	Mean	1.729422251
	SD	0.110709424

Source Data 1 Data used in Fig. 1B

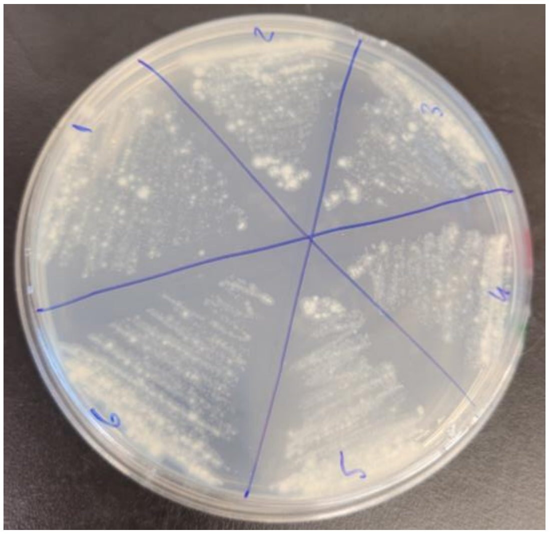

+ vapB5 + vapC5
(Quadrant labeled 6 was used)

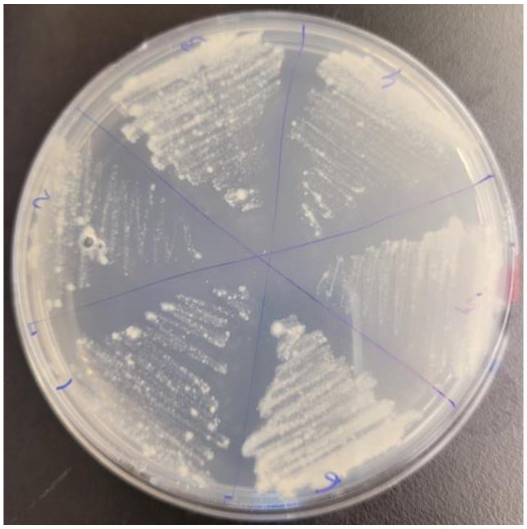

- vapB5 - vapC5
(Quadrant labeled 6 was used)

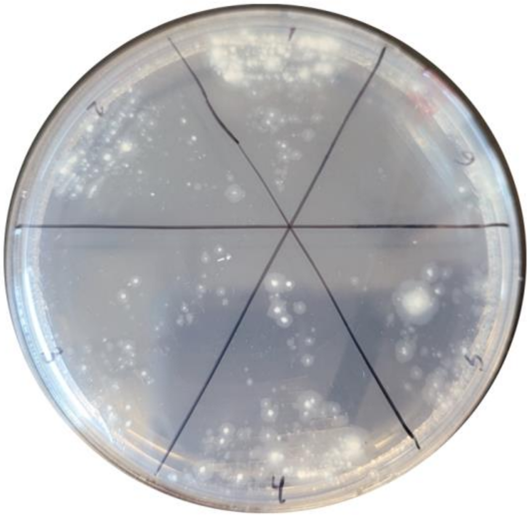

- vapB5 + vapC5
(Quadrant labeled 6 was used)

Source Data 2 Uncropped images from Fig. 1C

Time (hours)	Empty Vector			VapC5		
0	0.103	0.08	0.062	0.064	0.042	0.076
4	0.303	0.305	0.234	0.092	0.1	0.166
6	0.368	0.392	0.316	0.11	0.101	0.132
8	0.688	0.588	0.596	0.128	0.153	0.153
12	0.744	0.791	0.726	0.128	0.108	0.108
24	2.25	2.29	1.59	0.113	0.08	0.118

Source Data 3 Data used in Fig. 1D

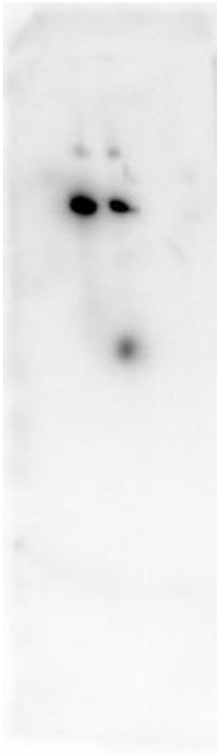

tRNA^{fMet}

tRNA$^{Ser\ CGA}$

Source Data 4. Uncropped images from Fig. 2B

	SigA	Tap	WhiB7	Erm(41)	Eis2	aac(2')
Control 2	21.391	31.971	27.401	25.733	21.145	22.726
Control 3	22.410	34.828	29.673	26.513	22.072	23.220
Control 4	22.213	33.207	28.312	26.267	21.275	23.703
Control 5	22.038	34.205	29.002	26.363	22.101	23.306
ATC 1	22.906	27.711	21.398	22.336	19.865	23.086
ATC 2	22.280	26.358	19.979	21.436	18.359	22.209
ATC 3	22.970	29.609	21.390	22.720	20.318	23.340
ATC 5	22.581	28.168	20.283	22.194	19.874	23.399
Control 2		10.581	6.011	4.342	-0.246	1.335
Control 3		12.418	7.263	4.102	-0.338	0.810
Control 4		10.993	6.099	4.054	-0.939	1.489
Control 5		12.167	6.964	4.325	0.063	1.268
ATC 1		4.805	-1.508	-0.570	-3.040	0.181
ATC 2		4.078	-2.301	-0.844	-3.921	-0.071
ATC 3		6.639	-1.580	-0.250	-2.652	0.370
ATC 5		5.586	-2.298	-0.387	-2.707	0.818
Control Average		11.540	6.584	4.206	-0.365	1.226
dd Ct 1		-6.734	-8.092	-4.775	-2.675	-1.045
dd Ct 2		-7.462	-8.885	-5.050	-3.556	-1.296
dd Ct 3		-4.901	-8.164	-4.456	-2.287	-0.855
dd Ct 5		-5.953	-8.882	-4.593	-2.342	-0.408
Fold Change 1		106	273	27	6	2
Fold Change 2		176	473	33	12	2
Fold Change 3		30	287	22	5	2
Fold Change 5		62	472	24	5	1
Log 2 FC 1		6.73440	8.09219	4.77549	2.67548	1.04501
Log 2 FC 2		7.46152	8.88536	5.05018	3.55632	1.29646
Log 2 FC 3		4.90078	8.16405	4.45588	2.28675	0.85548
Log 2 FC 5		5.95336	8.88194	4.59265	2.34212	0.40757
Mean		6.26	8.51	4.72	2.72	0.90
SD		1.10	0.44	0.26	0.59	0.38

Source Data 5 Data used in Fig. 3B
